# Inhibiting cGAS‐STING to Preserve Mitochondrial–Nuclear Communication and Stemness in Young Tendon Stem Cells: A Hydrogel Strategy against Age‐Related Tendinopathy

**DOI:** 10.1002/advs.202520941

**Published:** 2026-03-02

**Authors:** Zhuo Zhang, Weiyong Song, Heng Yin, Yi Wang, Yi Zou, Yuling Li, Yue Luo, Chao Xiang, Xiaoqin Liu, Yan Xiong, Yong Wang, Ke Jiang

**Affiliations:** ^1^ Department of Orthopedics Affiliated Hospital of North Sichuan Medical College Nanchong City Sichuan Province P. R. China; ^2^ Department of Dermatology Luzhou People's Hospital Luzhou City Sichuan Province P. R. China; ^3^ Department of Orthopaedics Daping Hospital, Army Medical University Chongqing City P. R. China; ^4^ Department of Orthopaedics The Affiliated Hospital of Southwest Medical University Luzhou City Sichuan Province P. R. China

**Keywords:** age‐related tendinopathy, hydrogel therapy, reactive oxygen species (ROS), mitochondria–nucleus communication, tendon‐derived stem cells (TDSCs)

## Abstract

Age‐related tendinopathy is common in the elderly. Their refractory nature is linked to low cellular density and poor blood supply of tendons. Key pathological features in aged tendons include the accumulation of senescent tendon‐derived stem cells (TDSCs), a decrease in young TDSCs, and an imbalance in the inflammatory microenvironment caused by reactive oxygen species (ROS). Among these, impaired mitochondria–nucleus communication is a central mechanism in disease progression. This study develops a ROS‐responsive dual‐targeted hydrogel (P/H@Lipo) loaded with selenium nanocatalysts (HPSe) and the STING inhibitor H‐151 in liposomes (L‐Lipo@H‐151). This system releases L‐Lipo@H‐151 in response to ROS within the inflammatory environment, targeting it to TDSCs to inhibit the cGAS‐STING pathway. The simultaneously released HPSe effectively reduces mtDNA leakage and cGAMP production, thereby strengthening the blockade of the cGAS‐STING pathway. This process maintains mitochondrial–nuclear communication, which in turn preserves the stemness of young TDSCs by preventing their senescence. Mechanistic studies indicate that HPSe boosts self‐renewal and tendinogenic differentiation in young TDSCs by inhibiting the Hippo signaling pathway. In summary, this study develops a novel therapeutic paradigm that targets the mitochondrial–nuclear communication to combat age‐related tendinopathy.

## Graphical abstract

**A dual‐targeting strategy to rejuvenate aged tendons**. A reactive oxygen species (ROS)‐responsive hydrogel co‐delivers a selenium nanozyme (scavenges ROS) and a STING inhibitor to tendon stem cells. This combined action restores mitochondria–nucleus communication, alleviates cellular senescence, and rejuvenates tendon regeneration, offering a novel therapy for age‐related tendinopathy.

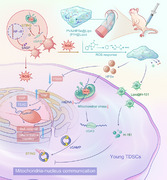

## 1 Introduction

With the increasing population of elderly individuals, research into prevention of age‐related diseases have attracted increasing interest. Achilles tendinopathy, a common primary degenerative tendon disorder [1], affects approximately 3%–26% of younger individuals; however, its incidence increases to 30%–50% in older adults [2, 3]. Patients experience localized tendon pain, functional impairment, and limitation of activity, especially during movement. Microscopically, there is persistent chronic edema and inflammatory infiltration within the tendon tissue [4]. Current clinical treatments mainly include local corticosteroid injections and surgical removal of affected tendons; however, these approaches are limited by uncertain effectiveness, significant invasiveness, and poor prognosis. Therefore, there is an urgent need to develop effective therapies for age‐related tendinopathy [5].

Tendon‐derived stem cells (TDSCs), a type of stem cell within tendon tissue with strong tendogenetic differentiation and self‐renewal abilities, serve as the main source for maintaining tendon health [6, 7]. In age‐related tendinopathies, TDSCs, the primary repair cells, significantly decline in number and mainly show signs of aging. Their ability to proliferate, differentiate, and promote tendinogenesis is greatly reduced [8], while they continue to release factors associated with cellular aging, known as senescence‐associated secretory phenotype (SASP). This induces macrophages in the surrounding environment to shift toward a pro‐inflammatory M1 state, fueling chronic low‐level inflammation in tissues [9]. When faced with trauma or excessive stress, this inflammatory response worsens rapidly, accelerating the aging and death of the already limited young TDSCs and severely limiting their ability to self‐renew and generate new tendon tissue. This is a key factor underlying the persistent problems encountered in age‐related tendinopathies [10, 11, 12]. Recent studies mainly focus on controlling the inflammatory environment and reprogramming macrophages to improve TDSC function [13, 14]. However, how to simultaneously regulate TDSC aging, eliminate reactive oxygen species (ROS), and reprogram macrophages to restore balance in aged tendons remains an urgent scientific challenge.

Mitochondria, as the core of cellular energy metabolism, play a pivotal role in the pathological process of age‐related tendinopathy. Impaired mitochondrial–nuclear communication in TDSCs is one of the core pathological mechanisms underlying this disease. In the inflammatory microenvironment, increased levels of ROS damage mitochondrial structure and function in young TDSCs, causing mitochondrial DNA (mtDNA) to leak into the cytoplasm and trigger the activation of the cGAS‐STING signaling pathway. This abnormal activation of cGAS‐STING disrupts normal mitochondrial–nuclear communication, leading to the activation of the nuclear NF‐κB pathway, the expression of SASP factors, and ultimately the senescence of young TDSCs [11, 15]. This process worsens local inflammation, creating a vicious cycle of “inflammation‐mitochondrial damage‐TDSC senescence‐inflammation escalation” that continually diminishes the already limited pool of young TDSCs. Therefore, this study proposes that targeting mitochondrial–nuclear communication, especially by inhibiting the cGAS‐STING pathway, could break this cycle and provide a new therapeutic approach for the management of age‐related tendinopathy.

Nanozymes are a class of nanomaterials with enzyme‐like catalytic functions that can catalyze specific biochemical reactions by mimicking the structure–function relationships of natural enzymes [16]. Among these, elemental selenium nanoparticles (SeNPs) demonstrate significant potential in treating age‐related tendinopathies due to their excellent biocompatibility, low toxicity, and potent antioxidant activity [17]. They directly undergo redox reactions with ROS in vitro, simultaneously scavenging ROS and mediating the reprogramming of macrophages toward an anti‐inflammatory phenotype [18], thereby offering a novel pathway to disrupt the vicious cycle of “inflammation‐aging.” In recent years, polyvinyl alcohol (PVA)‐based hydrogels have emerged as ideal carriers for delivery system design due to their excellent biocompatibility and tunable mechanical properties [19]. PVA, rich in hydroxyl (–OH) groups, can dynamically crosslink with phenylboronic acid (–PBA) groups via boronic acid esters, enabling three key functions: ROS‐responsive drug release, self‐healing, and injectability [20]. This smart responsiveness provides a vital technological foundation for the synergistic delivery of SeNPs and targeted drugs.

**SCHEME 1:**
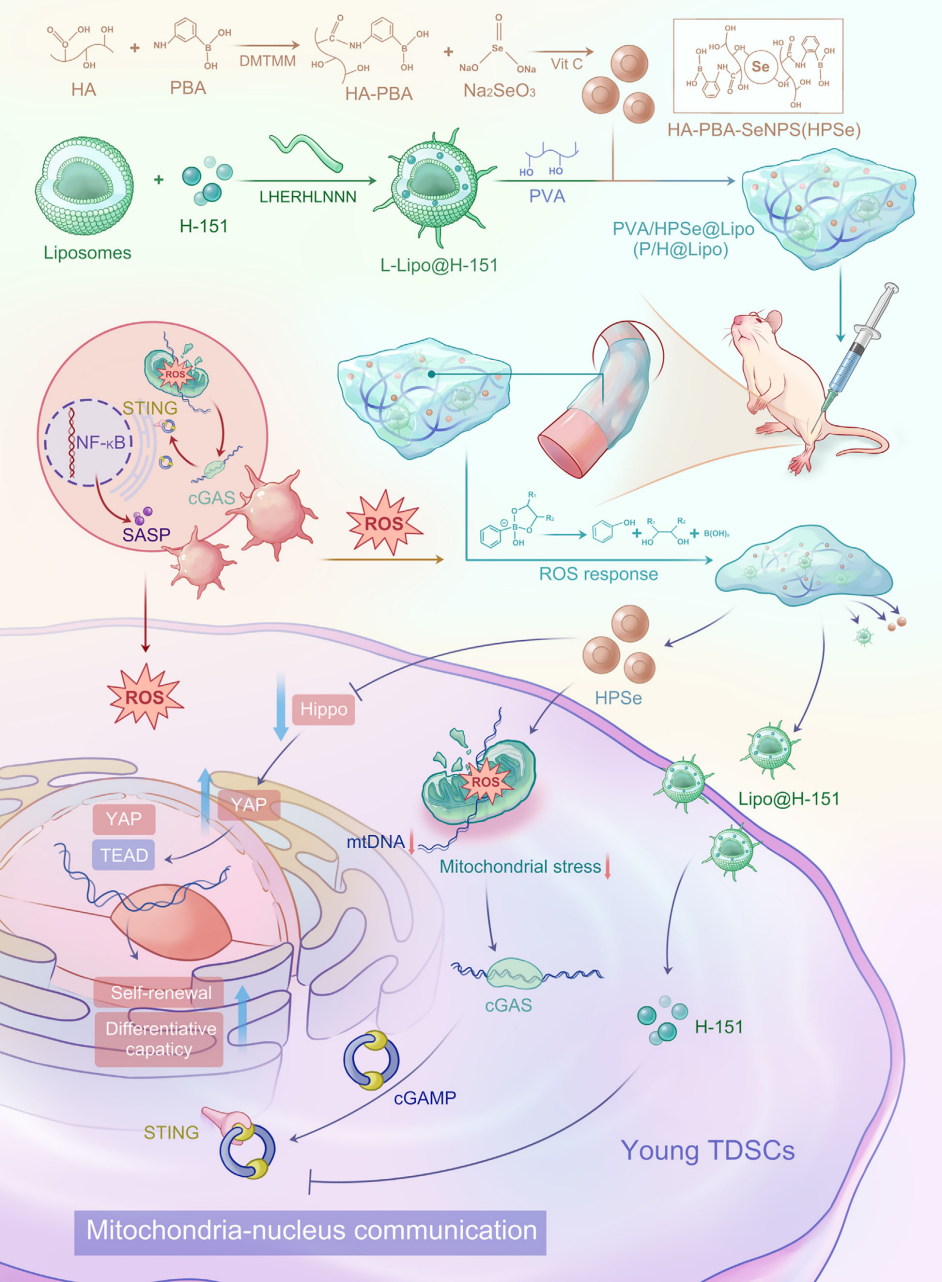
ROS‐responsive dual‐targeting hydrogel (PVA/HPSe@Lipo) modulates mitochondria–nucleus communication in young TDSCs for the treatment of age‐related tendinopathy.

This study aimed to develop an ROS‐responsive dual‐targeting hydrogel system (P/H@Lipo) to treat age‐related tendinopathy by synergistically regulating mitochondrial–nuclear communication (Scheme 1). This system was designed to create a gel network by dynamically crosslinking polyvinyl alcohol (PVA) with boronic acid esters and hyaluronic acid grafted with 3‐aminophenylboronic acid (HA‐PBA), while simultaneously loading the following: ① CD44‐targeted selenium nanocatalysts (HA‐PBA‐SeNPs) to scavenge ROS, mediate macrophage anti‐inflammatory reprogramming, and prevent mitochondrial DNA (mtDNA) leakage; and ② Type I collagen peptide (LHERHLNNN)‐modified liposomes (L‐Lipo@H‐151) for targeted delivery of the STING inhibitor H‐151 to block the cGAS‐STING pathway. In the tendon inflammatory microenvironment, P/H@Lipo responds to ROS to precisely release active components: the LHERHLNNN peptide guides liposomes to efficiently target TDSCs, while HA‐PBA‐SeNPs achieve secondary targeting via CD44‐mediated internalization to eliminate intracellular and extracellular ROS and reverse inflammation. Both agents work synergistically to inhibit the STING pathway through HPSe‐mediated suppression of mtDNA leakage and H‐151 blockade, bidirectionally regulating mitochondrial–nuclear communication. This results in the downregulation of nuclear senescence protein expression, significantly delaying aging in young TDSCs and promoting tendon regeneration, thus establishing a novel targeted delivery, multi‐pathway synergistic therapeutic strategy for age‐related tendinopathies.

## 2 Results and Discussion

### 2.1 Functional Differences between Aged and Young TDSCs

**FIGURE 1:**
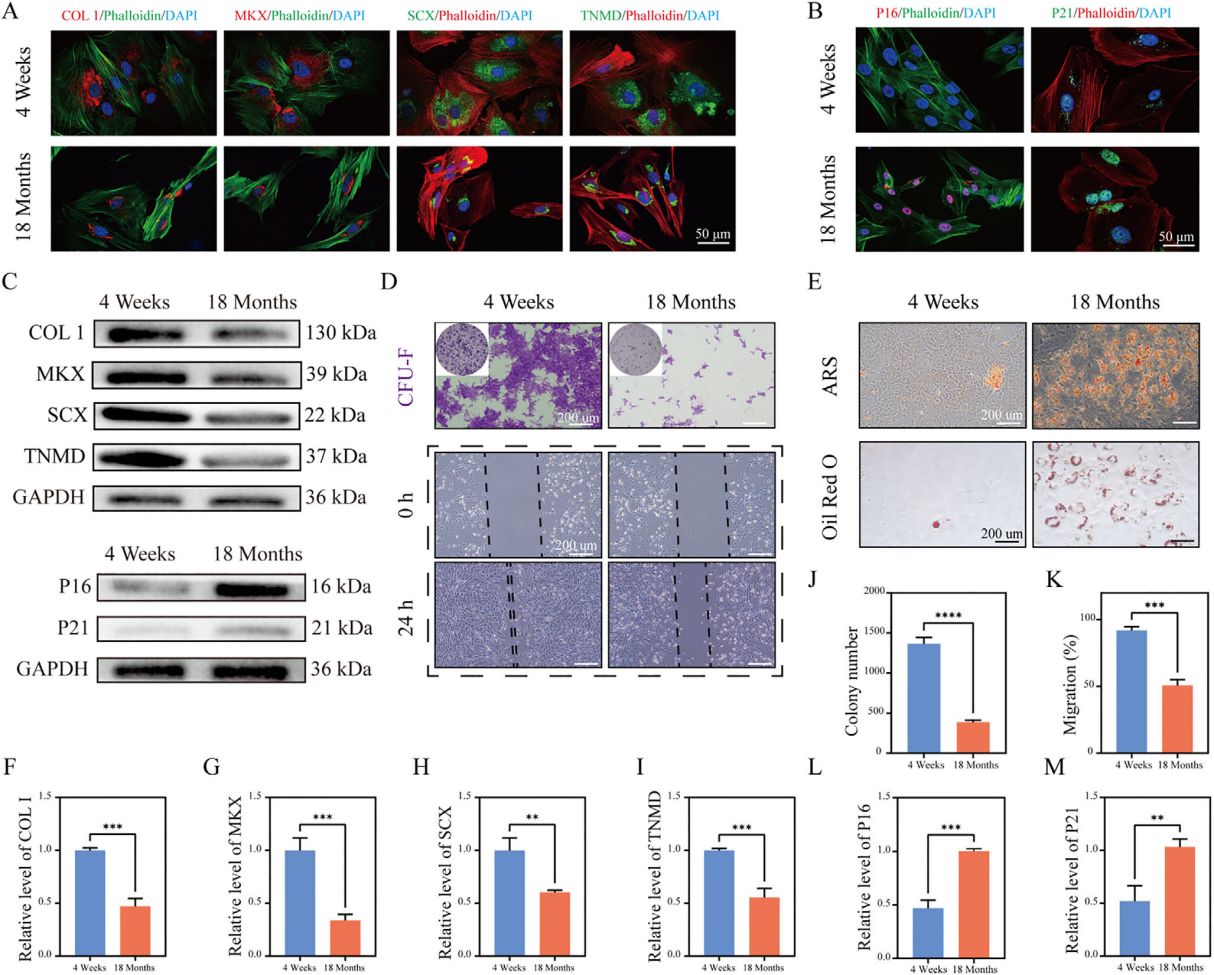
Functional differences between tendon‐derived stem cells (TDSCs) from aged and young tendons. A) Immunofluorescence images of tendon markers (COL I, TNMD, MKX, SCX) in TDSCs from 4‐week‐old and 18‐month‐old sources. Nuclei stained with DAPI; cytoskeleton stained with Phalloidin. B) Immunofluorescence images of senescence markers (P16, P21) in TDSCs. Nuclei stained with DAPI; cytoskeleton stained with Phalloidin. C) Western blot images showing levels of COL I, TNMD, MKX, SCX, P16, and P21 in TDSCs. D) Plate cloning assay and scratch assay of TDSCs. Plate cloning assay cells stained with crystal violet. E) Alizarin red and Oil Red O staining of TDSCs after 2 weeks of osteogenic and adipogenic induction. F–I) Semi‐quantitative analysis of COL I, TNMD, MKX, and SCX fluorescent proteins. J) Quantitative analysis of colonies in the plate cloning assay. K) Quantitative analysis of cell migration ratios in the TDSC scratch assay. L,M) Semi‐quantitative analysis of fluorescent proteins in P16 and P21 (*n* = 3 per group). Data are expressed as mean ± SD (*p < 0.05; **p < 0.01; ***p < 0.001).

Maintaining the stemness of young TDSCs is especially important for age‐related tendinopathies. To explore this, we compared functional differences between TDSCs from aged tendons (18 months old) and young tendons (4 months old). Immunofluorescence staining showed that the fluorescence intensity of four tendinogenesis‐related proteins—COL I, MKX, SCX, and TNMD—was significantly lower in aged TDSCs compared to young TDSCs (Figure 1A,F–I). Conversely, the fluorescence intensity of aging marker proteins P16 and P21 was notably higher in the aged group than in the young group (Figure 1B,L–M). Protein immunoblotting further confirmed this trend: young TDSCs expressed high levels of COL I, MKX, SCX, and TNMD, while aged TDSCs exhibited markedly increased P16 and P21 protein levels (Figure 1C, Figure S2). These findings indicate that aged TDSCs not only have reduced functional activity but also have higher levels of aging. Functionally, both colony formation and scratch assays demonstrated that young TDSCs had superior proliferation and migration abilities compared to aged TDSCs (Figure 1D,J,K). In addition, after 2 weeks of osteogenic and adipogenic differentiation induction, young TDSCs formed significantly fewer calcific nodules and lipid droplets compared to aged TDSCs (Figure 1E), indicating a lower tendency for abnormal differentiation and a better capacity to maintain tendinogenic differentiation.

TDSCs are essential for repairing tendon injuries due to their strong ability for self‐renewal and tendinogenic differentiation. This study systematically compared the functional capacities of TDSCs from aged and young tendons. Our findings showed that aged TDSCs exhibited decreased levels of proteins crucial for tendon health, including the main tendon component, type I collagen (COL I), as well as key developmental regulators Mohawk (MKX), Scleraxis (SCX), and the tendon differentiation‐promoting protein TNMD. Conversely, the expression of aging markers P16 and P21 was significantly higher. Functional tests further confirmed that aged TDSCs have reduced proliferation and migration capabilities and tend to undergo abnormal osteogenic and adipogenic differentiation. These findings suggest that the decline in function and pathological transdifferentiation of aged TDSCs are key factors in the failure of tendon regeneration. Therefore, developing strategies to delay aging in young TDSCs and maintain their stemness and functionality will be crucial for treating age‐related tendinopathies.

### 2.2 Synthesis and Characterization of HPSe

**FIGURE 2:**
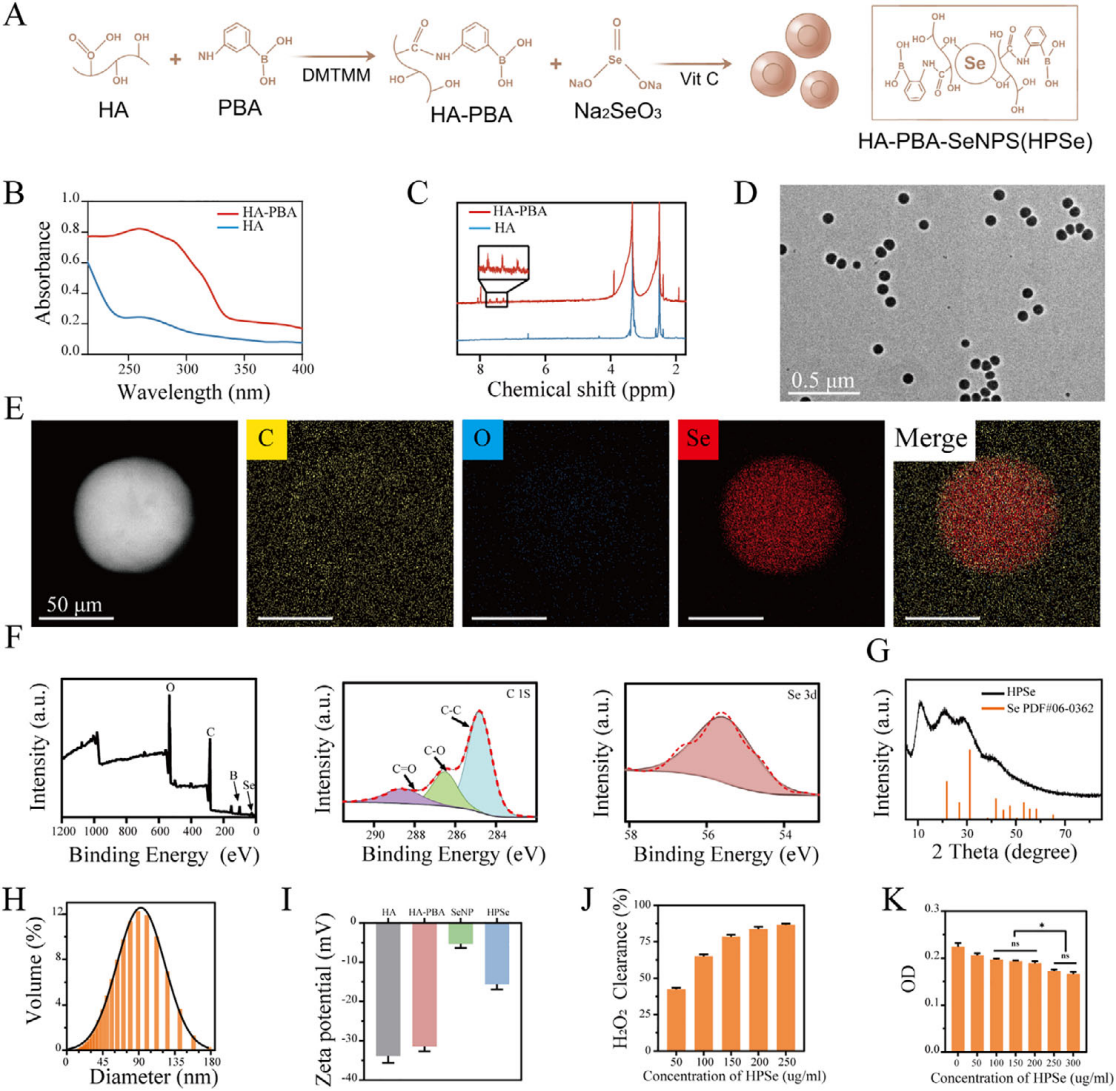
Synthesis and characterization of HPSe. A) Schematic diagram of HPSe synthesis. B) UV–vis spectroscopy of HA and HA‐PBA. C) Liquid NMR spectroscopy of HA and HA‐PBA. D) Transmission electron microscopy showing the size and morphology of HPSe. E) TEM mapping showing the elemental composition of HPSe. F) X‐ray photoelectron spectroscopy (XPS) of HPSe, including full spectrum and peak fitting of C 1s and Se 3d orbitals. G) X‐ray powder diffraction (XRD) of HPSe, including the standard pattern for Se. H) Hydrated particle size distribution of HPSe. I) Zeta potential of HA, HA‐PBA, SeNP, and HPSe. J) Hydrogen peroxide scavenging assay of HPSe. K) CCK‐8 assay after co‐culture of TDSCs with HPSe (*n* = 3 per group) Data are expressed as mean ± SD (*p < 0.05; **p < 0.01; ***p < 0.001).

In this study, we first activated the carboxyl group (–COOH) of hyaluronic acid (HA) using DMTMM, enabling its reaction with phenylboronic acid (PBA) to synthesize the HA‐PBA complex [21]. Subsequently, ascorbic acid (Vc) was employed to reduce sodium selenite (Na_2_SeO_3_) in the HA‐PBA solution, successfully yielding HPSe nanoparticles [22]. The synthesis of HA‐PBA was first validated by analyzing HA and HA‐PBA using UV spectrophotometry. The results revealed a distinct absorption peak for HA‐PBA near 280 nm (Figure 2A), corresponding to the characteristic absorption peak of the benzene ring and confirming successful grafting of PBA onto HA. Further characterization of HA‐PBA via ^1^H NMR spectroscopy detected benzene ring absorption peaks at 7.2–7.8 ppm. Calculations based on the characteristic HA methyl peak at 1.89 ppm yielded a grafting rate of approximately 68%, further confirming the successful synthesis of HA‐PBA (Figure 2B).

Transmission electron microscopy (TEM) images show that HPSe has a uniform spherical distribution with a particle size of about 82 ± 0.56 nm (Figure 2C). Elemental mapping analysis indicated that its composition included C, O, and Se (Figure 2D). Scanning electron microscopy images and point scanning results similarly reveal spherical HPSe particles containing C, N, O, and Se (Figure S3A,B). Full‐spectrum X‐ray photoelectron spectroscopy (XPS) analysis displays characteristic peaks for C, O, and Se at 280, 180, and 56 eV, respectively (Figure 2E). High‐resolution C 1s spectrum fitting shows bonding states at 288.3 eV (C═C), 287.2 eV (C–O), and 284.6 eV (C–C), consistent with the HA‐PBA structure. The peak at 55.8 eV in the Se 3d spectrum confirmed the presence of elemental selenium, verifying the successful reduction of Na_2_SeO_3_. The diffraction peaks at 2θ = 23.4° and 29.6° in the X‐ray diffraction (XRD) pattern matched the standard card for elemental Se (PDF No. 06‐0362) (Figure 2F).

Dynamic light scattering (DLS) analysis showed that the hydrated particle size of HPSe was approximately 90 ± 0.21 nm, with a polydispersity index (PDI) of approximately 0.2 (Figure 2G), indicating excellent particle dispersion. After incubation at different time points in PBS containing 10% serum (pH = 7.4), no significant changes in particle size were observed, demonstrating good in vitro stability (Figure S4C). Zeta potential measurements showed potentials of −33 mV for HA, −30 mV for HA‐PBA, −5 mV for SeNPs (selenium nanoparticles without stabilizer), and −15 mV for HPSe (Figure 2H). This confirmed that HA‐PBA modification effectively improves the stability of selenium nanoparticles.

The hydrogen peroxide scavenging assay was used to assess the ability of HPSe to eliminate ROS. As the concentration of HPSe increased, the scavenging rate of H_2_O_2_ steadily increased, reaching 82.4% at 200 µg/mL (Figure 2I). The CCK‐8 cytotoxicity assay showed that TDSC activity significantly decreased at a concentration of 250 µg/mL (Figure 2J). Given its H_2_O_2_ (ROS) scavenging ability and cellular compatibility, 200 µg/mL was chosen as the working concentration of HPSe for subsequent experiments in this study.

This study synthesized selenium nanoparticles (HPSe) stabilized using HA‐PBA. Studies indicate that pure selenium nanoparticles (SeNPs) possess high surface energy; therefore, they readily agglomerate in solutions, leading to a reduction in their antioxidant activity and drug‐release properties [23]. To address this issue, capping agents are often added to prevent nanoparticle overgrowth and improve dispersion stability. Common capping agents include polyvinylpyrrolidone (PVP), polyethylene glycol (PEG), and hyaluronic acid (HA). Based on the target cell mechanism and the requirements for hydrogel construction, this study selected grafted PBA‐HA (HA‐PBA) as the SeNP capping agent, thereby preparing HPSe by forming Se–O bonds. This composite material maintains the ROS scavenging and macrophage reprogramming functions of SeNPs while showing excellent dispersibility. It can also form hydrogels through crosslinking with PVA through borate bonds.

Ultraviolet spectroscopy and ^1^H NMR confirmed the successful grafting of PBA onto HA. XRD, XPS, TEM, and elemental mapping analyses collectively validated the successful synthesis of HPSe. Particle size distribution and H_2_O_2_ scavenging experiments demonstrated HPSe's excellent stability and significant ROS scavenging capacity. Cell compatibility was assessed using CCK‐8 assays, which identified the optimal concentration for subsequent experiments.

Furthermore, HA is often used in constructing various targeted nanomaterials because of its excellent biocompatibility and ability to specifically bind to the CD44 receptor on cell membranes [24, 25]. In the HPSe synthesized in this study, the conformation of HA remains unchanged, thereby preserving its capacity to target CD44‐positive cells. Previous studies reported that TDSCs and macrophages express high levels of CD44 [26, 27], indicating that HPSe has the potential for targeted delivery to these two cell types. Future research will further verify the targeting ability of HPSe within hydrogel systems through cellular uptake experiments.

### 2.3 Synthesis, Characterization, and ROS Responsiveness of Liposome and Hydrogel Systems Using Dual‐Targeting Validation

**FIGURE 3:**
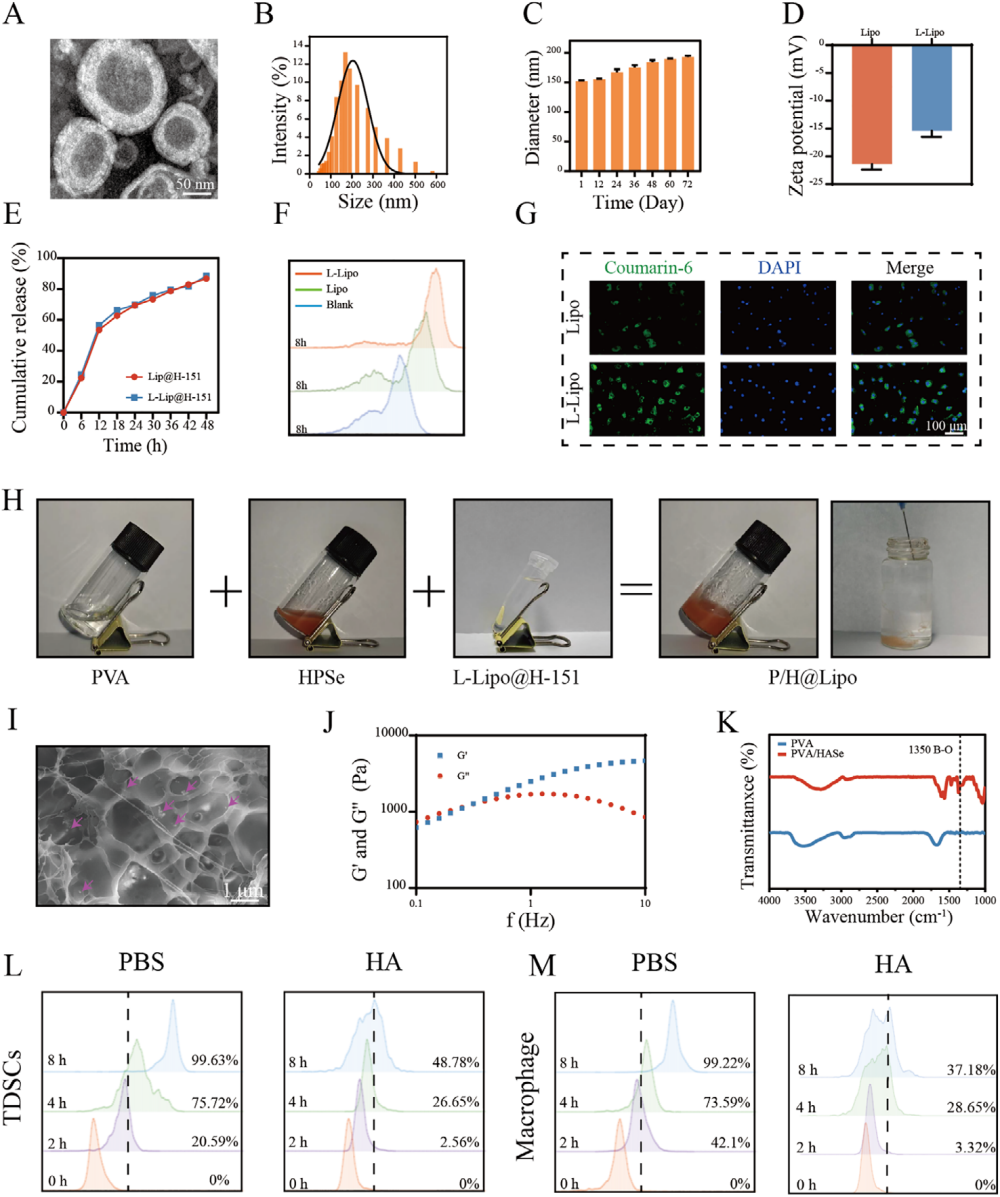
Synthesis, characterization, and dual‐targeting validation of liposomes and hydrogel systems. A) Transmission electron microscopy (TEM) images showing liposome size and morphology. B) Liposome particle size distribution. C) In vitro stability of liposomes. D) Zeta potential of liposomes. E) H‐151 release profiles from liposomes before and after grafting with targeting peptides. F,G) Flow cytometry and fluorescence microscopy detection of liposome targeting to TDSCs. H) Synthesis and injectability validation of the hydrogel system. I) Scanning electron microscopy showing the morphology of the lyophilized hydrogel system (pink arrows indicate selenium nanoparticles). J) Rheological testing of the hydrogel system. K) Infrared spectra comparison between PVA and the hydrogel system (dashed line indicates characteristic peak at 1350 cm^−^^1^ for B–O bond). L–O) Flow cytometry analysis of TDSCs co‐cultured with HA solution (with or without macrophage sealing) and the hydrogel system at different time points (green: coumarin 6‐labeled liposomes; nuclei stained with DAPI; cytoskeleton stained with phalloidin) (*n* = 3 per group). Data are expressed as mean ± SD (*p < 0.05; **p < 0.01; ***p < 0.001).

This study initially characterized the synthesized targeted peptide liposomes. TEM images revealed that the liposomes displayed a typical bilayer spherical structure with a particle size of approximately 155.21 ± 0.78 nm (Figure 3A). UV spectrophotometry showed that the drug encapsulation efficiency of H‐151 in the liposomes was approximately 82.7%. DLS results indicated an average particle size of approximately 161.3 ± 0.47 nm and a PDI of about 0.17, confirming uniform dispersion. After 12 days of incubation in PBS containing 10% serum (pH = 7.4), the particle size gradually increased to approximately 194.37 ± 0.52 nm, indicating good stability (Figure 3B, C). Zeta potential measurements revealed a shift from −21.5 to −14.8 mV before and after targeting peptide modification (Figure 3D). Drug release experiments demonstrated sustained release of H‐151 over 48 h, with a cumulative release rate of 88.5%, indicating that targeting peptide modification did not affect drug release behavior (Figure 3E). Further analysis using coumarin 6‐fluorescently labeled liposomes, flow cytometry, and immunofluorescence confirmed that liposomes modified with the targeting peptide exhibited significantly enhanced targeting capacity toward TDSCs within 8 h (Figure 3F,G).

Direct mixing of PVA, HPSe, and L‐Lipo@H‐151 causes gelation. The resulting composite hydrogel can be smoothly extruded from a hypodermic needle into an aqueous phase while maintaining its gel state, demonstrating excellent injectability (Figure 3H). Scanning electron microscopy images show that the freeze‐dried hydrogel (PVA/HPSe@Lipo) has a porous structure, with selenium nanoparticles (indicated by red arrows) distributed throughout the matrix (Figure 3I). Rheological tests of the composite hydrogel reveal that the storage modulus (*G*′) consistently exceeds the loss modulus (*G*″) with increasing frequency, indicating sustained gelation. Fourier transform infrared (FTIR) spectroscopy detects a characteristic absorption peak at 1350 cm^−^^1^ for the B–O bond, confirming the presence of borate linkages. These results indicate that PVA/HPSe@L‐Lipo exhibits structural stability through borate ester crosslinking (Figure 3J,K).

To evaluate the ROS responsiveness of the composite hydrogel system, PVA/HPSe@L‐Lipo samples were placed in PBS and H_2_O_2_ solutions, respectively, and selenium release behavior was measured using UV spectrophotometry. Our results showed significantly higher selenium release in the H_2_O_2_ group compared to the PBS group, indicating that the borate ester bonds in the composite hydrogel system undergo cleavage under oxidative conditions, enabling ROS‐responsive release (Figure S4D). Cell viability staining of the composite hydrogel system demonstrated its excellent biocompatibility, making it suitable for subsequent experiments (Figure S4E,F).

Furthermore, coumarin 6 was used to fluorescently label HPSe and prepare a composite hydrogel system. This system was co‐cultured with TDSCs and macrophages, respectively, followed by flow cytometry and immunofluorescence analysis at 0, 2, 4, and 8 h. Our results showed that the uptake rate of HPSe by TDSCs and macrophages significantly increased over time, reaching approximately 99% after 8 h. However, pretreatment with HA solution to block CD44 resulted in uptake rates below 50% after 8 h. This demonstrates that HPSe exhibits excellent specific targeting ability toward cells highly expressing CD44 (Figure 2L,M and S4G).

This study developed a ROS‐responsive dual‐targeted hydrogel system (P/H@Lipo) capable of simultaneously loading selenium nanocatalysts (HPSe) and STING inhibitor H‐151‐encapsulated targeted liposomes (L‐Lipo@H‐151). Experiments show that this hydrogel, formed through dynamic borate crosslinking, enables sustained release of HPSe and L‐Lipo@H‐151 for over 2 weeks. This timeframe aligns with the therapeutic cycle for myositis, providing excellent temporal control.

Liposomes, as a traditional drug delivery system, effectively encapsulate and protect drug molecules through their phospholipid bilayer, prolonging drug half‐life with sustained release. However, conventional liposomes lack targeting ability, which limits delivery efficiency. To overcome this, we modified the liposome surface with a type I collagen‐specific binding peptide (LHERHLNNN). This peptide binds specifically to type I collagen [28], which is highly expressed and secreted by young TDSCs, enabling active targeting of these cells and improving the efficacy of drug delivery. Target validation experiments further showed that L‐Lipo@H‐151 efficiently accumulates within young TDSCs.

Regarding the HPSe targeting mechanism, cellular uptake experiments demonstrated that both TDSCs and macrophages exhibited high uptake rates for HPSe. This uptake effect was significantly inhibited by HA pretreatment, confirming its dependence on CD44 receptor‐mediated endocytosis pathways. This finding aligns with the design of HA‐PBA, which retains its CD44 targeting capability.

Compared to most conventional hydrogels requiring surgical implantation, the P/H@Lipo system developed in this study exhibits excellent injectability and self‐healing capabilities. It enables minimally invasive drug delivery, reduces the risk of secondary tissue damage and infection, maintains structural integrity and functionality around tendons, and achieves sustained local drug release. Overall, this study successfully synthesized and systematically characterized an ROS‐responsive P/H@Lipo hydrogel system with dual‐targeting functionality, providing a solid experimental foundation for its further application in the treatment of tendinopathy.

### 2.4 P/H@Lipo Mitigates Aging in Young TDSCs by Regulating Mitochondrial–Nuclear Communication

**FIGURE 4:**
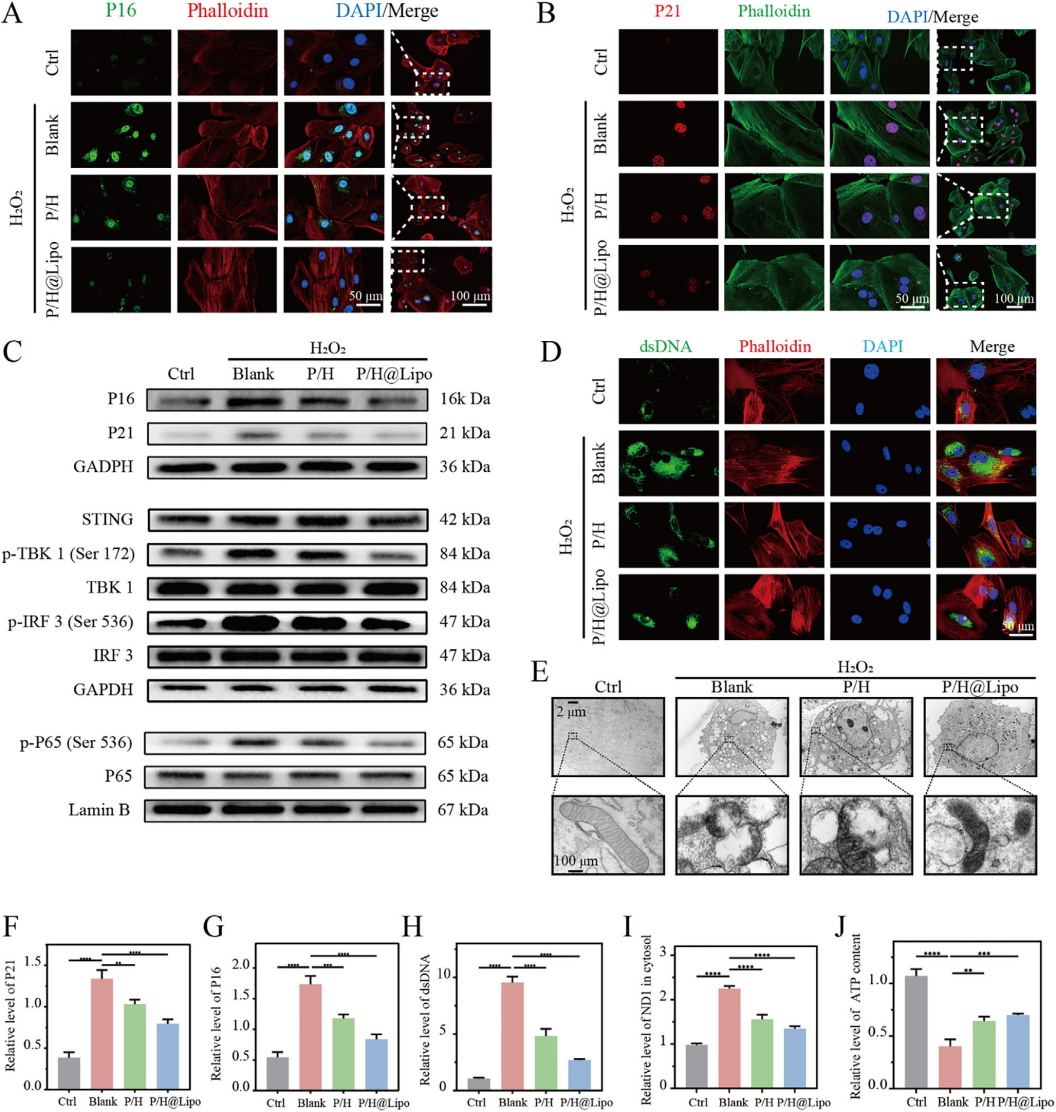
P/H@Lipo mitigates aging in young TDSCs by regulating mitochondrial–nuclear communication. A,B) P16 and P21 immunofluorescence staining in TDSCs. Nuclei stained with DAPI; cytoskeleton stained with phalloidin. C) Western blot images showing P16, P21, and STING pathway‐related protein levels in TDSCs. D) dsDNA immunofluorescence staining of TDSCs. E) Transmission electron microscopy of mitochondria in TDSCs. F–H) Semi‐quantitative analysis of immunofluorescence. I) Ratio of mtDNA to total DNA in TDSCs. J) Relative APT levels in TDSCs. Data are expressed as mean ± SD (*n* = 3 per group). Data are expressed as mean ± SD (*p < 0.05; **p < 0.01; ***p < 0.001).

To simulate the inflammatory microenvironment experienced by young TDSCs in aged tendons, this study employed a co‐culture system of aged and young TDSCs, utilizing H_2_O_2_ to mimic tendinopathy‐associated inflammatory stimuli. We established a comprehensive control system in the H_2_O_2_‐induced TDSC aging model, including: Ctrl, Blank, L‐Lipo@H‐151, HPSe, L‐Lipo@H‐151+HPSe, and P/H@Lipo. Evaluation was conducted using aging markers (P16/P21) and functional markers of TDSCs (COL 1) (Figure S5). Results showed that both monotherapies partially alleviated aging but with limited efficacy. In contrast, the physical mixture and P/H@Lipo groups demonstrated significantly stronger anti‐aging effects, with comparable potency. Regarding cellular function, both monotherapies partially mitigated H_2_O_2_‐induced functional suppression of TDSCs. However, the physical mixture and P/H@Lipo groups exhibited significantly stronger maintenance of TDSC function, again with comparable efficacy. These findings directly demonstrate the synergistic effect between HPSe and L‐Lipo@H‐151. Immunofluorescence staining and Western blot analysis revealed that H_2_O_2_ treatment significantly upregulated the expression of age‐related proteins P16 and P21 in young TDSCs. Co‐culture with P/H effectively mitigated this senescent phenotype, while combined treatment with H‐151‐loaded liposomes demonstrated enhanced anti‐senescent effects (Figure 4A,B,F,G). We further compared the anti‐aging effects of non‐targeted liposomes (Lipo@H‐151) and targeted liposomes (L‐Lipo@H‐151). As shown in Figure S6, both significantly reduced the expression of aging markers (P16/P21) more effectively than the Blank group, confirming the efficacy of the H‐151 drug. More importantly, the anti‐aging effect of the L‐Lipo@H‐151 group was significantly stronger than that of the Lipo@H‐151 group.

Protein immunoblotting analysis of the cGAS‐STING signaling pathway revealed that P/H (PVA/HPSe) treatment partially inhibited this pathway and significantly suppressed P65 phosphorylation, highlighting its potent inhibitory effect on the NF‐κB signaling pathway, consistent with the findings of previous studies. Following combined L‐Lipo@H‐151 treatment, the cGAS‐STING signaling pathway was markedly suppressed, with further decreased P65 phosphorylation levels. Concurrently, P16 and P21 expression was also significantly reduced (Figure 4C and S8A–F).

dsDNA immunofluorescence staining revealed that H_2_O_2_ treatment significantly increased dsDNA fluorescence intensity in the cytoplasm of young TDSC cells. P/H treatment markedly reduced this signal, with P/H@Lipo intervention demonstrating even more pronounced effects (Figure 4D). Transmission electron microscopy revealed that H_2_O_2_ induced mitochondrial swelling and membrane structural deformation, whereas P/H@Lipo treatment markedly improved mitochondrial morphology (Figure 4E).

Mitochondrial DNA (mtDNA) and total DNA (nDNA) in the cytoplasm were quantified using qPCR. The mtDNA/nDNA ratio in the H_2_O_2_ group was approximately 2.13, and decreased to 1.57 after P/H intervention and further decreased to approximately 1.47 after P/H@Lipo treatment (Figure 4I). ATP content detection results also indicated that P/H@Lipo intervention could increase ATP levels from approximately 0.47 to 0.71 (Figure 4J).

The combined results above demonstrate that P/H@Lipo effectively protects mitochondrial structure and function, reduces mtDNA leakage, and mitigates the aging process in young TDSCs within an inflammatory microenvironment. Although the synergistic effects of physical mixing and co‐assembly were comparable in vitro, in vivo tendinopathy treatment requires sustained drug retention at the lesion site and controlled release. Therefore, we ultimately selected a gel‐forming system for subsequent experiments to validate the therapeutic efficacy of this optimized system in complex physiological environments. The enhanced efficacy of L‐Lipo@H‐151, when combined with the evidence from Figure 3G that the targeting peptide significantly enhances liposome cellular uptake, collectively demonstrates that the targeted design improves drug delivery efficiency and therapeutic efficacy, making it a critical component in optimizing therapeutic systems.

The cGAS‐STING signaling pathway plays a pivotal role in autoimmune and chronic inflammatory diseases [29], being recognized as one of the core pathways in cellular stress responses. It detects endogenous or exogenous double‐stranded DNA (dsDNA) within the cytoplasm and links DNA damage to inflammatory responses. Specifically, cytoplasmic DNA (originating from either the nucleus or mitochondria) activates cGAS to catalyze the production of the second messenger cGAMP. Upon binding to the STING protein on the endoplasmic reticulum, cGAMP activates downstream TBK1, which subsequently phosphorylates IRF3 and the NF‐κB subunit P65. This ultimately induces the secretion of inflammatory cytokines and SASP factors, leading to cellular senescence [30, 31, 32]. mtDNA, as a circular double‐stranded DNA molecule within the mitochondrial matrix, is strictly confined to mitochondria under normal conditions. However, within the inflammatory microenvironment, impaired oxidative phosphorylation, metabolic dysfunction, and mitochondrial structural disruption in young TDSCs facilitate mtDNA leakage into the cytoplasm via mitochondrial permeability transition pores (mPTP) or Bak/Bax channels. This mtDNA is then recognized by cGAS, activating the cGAS‐STING signaling pathway, which drives senescence in young TDSCs.

Previous studies have demonstrated that inhibiting the STING signaling pathway can delay aging to a certain extent [33, 34]. However, in the context of age‐related tendinopathy, simply blocking STING fails to completely reverse the aging process in young TDSCs. This is because the persistent high ROS microenvironment means the source of mtDNA leakage remains unaddressed, potentially allowing the cGAS‐STING pathway to remain continuously activated. The P/H@Lipo hydrogel system developed in this study employs a dual‐mechanism synergistic intervention: First, it scavenges ROS and protects mitochondrial structure and function, thereby reducing mtDNA leakage at its source; second, it blocks signal transduction via the STING inhibitor H‐151. This combined action efficiently suppresses the cGAS‐STING pathway activation and restores mitochondrial‐nuclear communication, significantly alleviating the senescence phenotype in young TDSCs. Experimental results confirmed that this strategy effectively reduces the expression of aging markers, improves mitochondrial function, and restores ATP levels. The alleviation of aging phenotypes indicates enhanced cellular function. Subsequent studies will further investigate the ameliorative effects of P/H@Lipo on functional aspects such as myotendinous differentiation and migration in young TDSCs.

### 2.5 P/H@Lipo Alleviates Functional Suppression, DNA Damage, and Apoptosis in Young TDSCs Caused by Oxidative Stress

**FIGURE 5:**
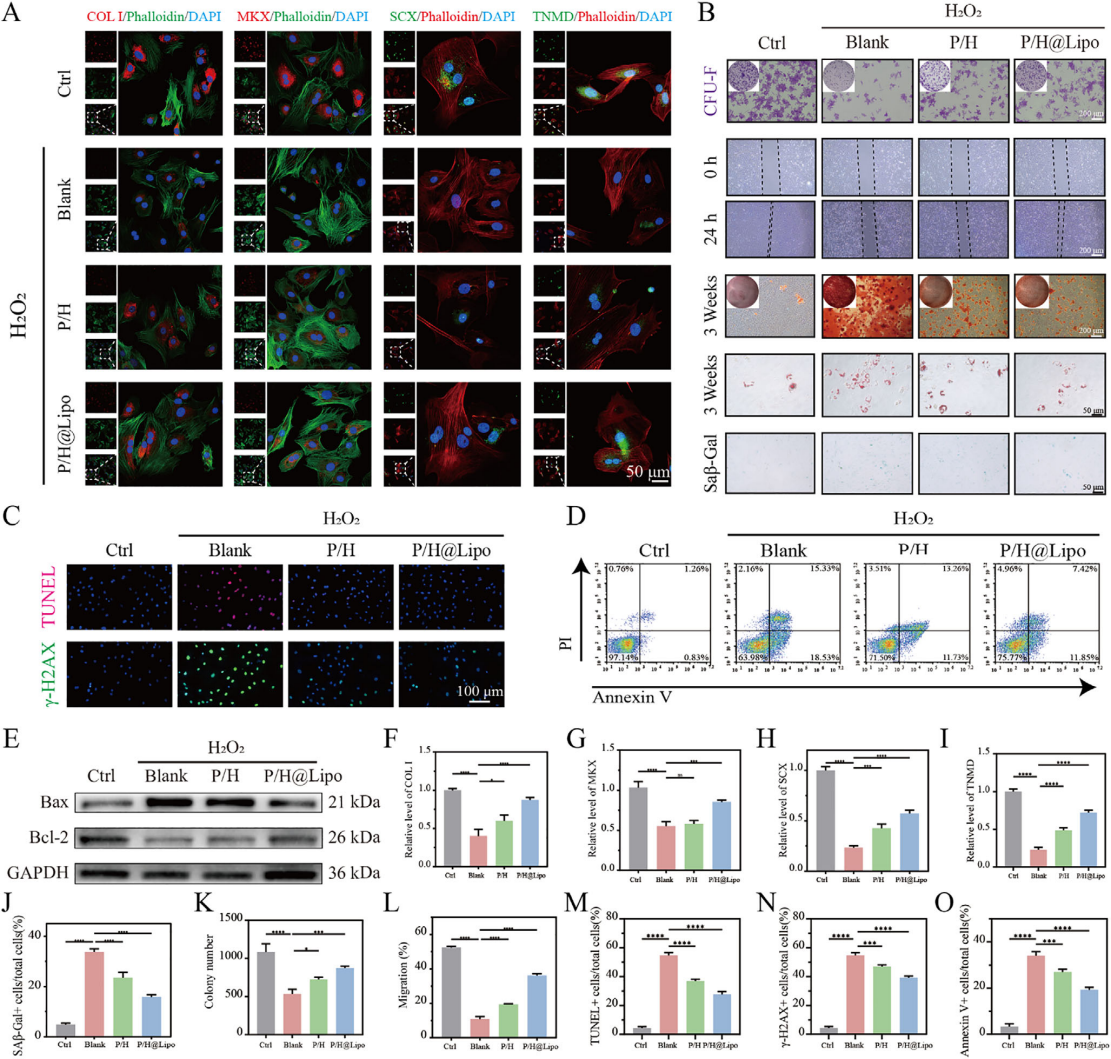
P/H@Lipo alleviates functional suppression, DNA damage, and apoptosis in young TDSCs caused by oxidative stress. A) Immunofluorescence staining images of tendon markers (COL I, TNMD, MKX, and SCX) in TDSCs. Cell nuclei were stained with DAPI and cytoskeleton stained with phalloidin. B) Plate cloning assay and scratch assay of TDSCs. Plate cloning assay cells stained with crystal violet. Alizarin red and Oil Red O staining of TDSCs after 2 weeks of osteogenic and adipogenic induction. β‐galactosidase staining of TDSCs. C) TUNEL staining and immunofluorescence staining for DNA damage‐associated protein γ‐H2AX. Cell nuclei stained with DAPI. D) Flow cytometric analysis after Annexin V/PI staining. E) Western blot images of Bax and Bcl‐2 proteins. F–I) Semi‐quantitative analysis of COL I, TNMD, MKX, and SCX fluorescent proteins. J) Ratio of β‐galactosidase‐positive cells. K) Quantitative analysis of colonies in the plate cloning assay. L) Quantitative analysis of cell migration ratio in scratch assay. M) TUNEL‐positive cell ratio. N) 𝛾‐H2AX‐positive cell ratio. O) Annexin V‐positive cell ratio (*n* = 3 per group). Data are expressed as mean ± SD (*p < 0.05; **p < 0.01; ***p < 0.001).

By assessing the expression levels of four proteins—COL I, MKX, SCX, and TNMD—the tendinogenic potential of young TDSCs can be evaluated. Immunofluorescence staining revealed that H_2_O_2_ treatment significantly downregulated the expression of these four functional proteins. Following ROS scavenging with P/H, their expression levels markedly rebounded, indicating that P/H effectively mitigated the impact of oxidative stress on cellular function. Further co‐culture with P/H@Lipo more markedly restored protein expression levels, suggesting that P/H@Lipo effectively improves the functional state of young TDSCs (Figure 5A,F–I). Under high ROS levels in the inflammatory microenvironment, young TDSCs are prone to DNA damage, which induces apoptosis and severely impairs tendon repair capacity. 𝛾‐H2AX immunofluorescence staining for DNA damage revealed that HPSe significantly mitigated H_2_O_2_‐induced DNA damage, with a more pronounced inhibitory effect when combined with anti‐aging interventions. TUNEL staining results demonstrated that HPSe significantly inhibited H_2_O_2_‐induced apoptosis, with the anti‐apoptotic effect further enhanced upon co‐administration of anti‐aging drugs (Figure 5C,M,N). Flow cytometry and Annexin V/PI assays quantitatively confirmed the following: the apoptosis rate in the H_2_O_2_ group was approximately 34%, reduced to 27% following HPSe intervention, and further decreased to around 18% when combined with anti‐aging liposome treatment (Figure 5F,O). Protein immunoblotting analysis of apoptosis‐related proteins Bax and Bcl‐2 revealed that H_2_O_2_ treatment significantly upregulated the pro‐apoptotic protein Bax and suppressed the expression of the anti‐apoptotic factor, protein Bcl‐2. Co‐culture with P/H partially inhibited this trend, while combined treatment with L‐Lipo@H‐151 significantly reversed it (Figure 5E). These findings conclusively demonstrate that the P/H@Lipo hydrogel system exhibits outstanding anti‐DNA damage and anti‐apoptotic effects, effectively preserving the functional activity of young TDSCs under oxidative stress conditions.

Most of the cells in aging tendon tissues have entered a senescent state, with only a small number of young TDSCs remaining crucial for maintaining tendon repair capacity. Prior to tendinopathy onset, senescent cells continuously secrete SASP factors, maintaining the tendon in a chronic state of low‐grade inflammation. This leads to a degree of functional suppression in young TDSCs. Once tendinopathy develops, the internal homeostasis of the aged tendon is disrupted, causing a sharp rise in local inflammation. This further inhibits the oxidative respiratory chain of young TDSCs, impairing mitochondrial structure and function. This not only exacerbates the dysfunction of young TDSCs but also induces DNA damage, senescence, and even apoptosis, ultimately significantly diminishing the tendon's repair capacity.

The preceding section has validated the pathological alterations in young TDSCs from the perspectives of aging phenotypes and mitochondrial function. This study further demonstrates, through functional protein expression analysis and DNA damage/apoptosis assays, that the P/H@Lipo hydrogel system effectively maintains the expression levels of key functional proteins—including COL I, MKX, SCX, and TNMD—in young TDSCs under ROS stimulation, significantly mitigating DNA damage and inhibiting cell apoptosis. This hydrogel system protects young TDSCs through multiple pathways, thereby reducing abnormal depletion. Consequently, it provides more functional repair cells for age‐related tendinopathy, significantly enhancing tissue self‐repair potential and offering an effective strategy for promoting tendon regeneration.

### 2.6 P/H@Lipo Enhances the Proliferation and Migration Capacity of Young TDSCs, Reduces Osteogenic and Adipogenic Differentiation, and Delays Senescence

The proliferation capacity of young TDSCs was assessed via plate cloning assays. Crystal violet staining revealed that H_2_O_2_ intervention reduced the number of cell clones from approximately 1068 to 561. Following HPSe treatment, the number recovered to approximately 726; however, intervention with the P/H@Lipo hydrogel system further increased the number to approximately 874, demonstrating that this system can effectively enhance the proliferation capacity of young TDSCs (Figure 5B,K). Scratch assay results revealed that H_2_O_2_ treatment reduced the 24‐h migration rate of young TDSCs from 54% to 16%; following HPSe intervention, the migration rate rebounded to 21%; whereas the P/H@Lipo‐treated group further increased to 38%, demonstrating that this system effectively promotes cell migration (Figure 5B,L).

Following 3 weeks of osteogenic/adipogenic induction differentiation, alizarin red and oil red O staining revealed that H_2_O_2_ significantly enhanced osteogenic and adipogenic differentiation in young TDSCs, whereas HPSe treatment markedly suppressed this effect. The inhibitory action was further amplified upon co‐treatment with anti‐aging agents, demonstrating that P/H@Lipo effectively curbs abnormal differentiation tendencies and preserves the cells’ tendinogenic functional orientation (Figure 5B). SA‐β‐galactosidase staining revealed that the proportion of H_2_O_2_‐induced senescent cells increased from 6% to 36%; following HPSe intervention, this decreased to 25%; however, P/H@Lipo treatment further reduced it to 17%, demonstrating that this hydrogel system effectively delays the senescence process in young TDSCs (Figure 5B).

Within the inflammatory microenvironment, the function of young TDSCs is significantly suppressed and progressively ages, primarily manifested as diminished proliferation and migration capabilities, directly compromising the intrinsic repair potential of aging tendons. As stem cells possessing multidirectional differentiation potential, TDSCs primarily differentiate towards the tendinous lineage in young, healthy tendon tissue. However, under the influence of aging and oxidative stress, their normal tendinogenic differentiation capacity diminishes, giving way to abnormal tendencies toward adipogenic and osteogenic differentiation. This severely compromises the quality of tendon tissue repair. This study demonstrates that the P/H@Lipo hydrogel system effectively maintains the proliferation and migration functions of young TDSCs, significantly preserves their tendinogenic differentiation capacity, and suppresses inflammation‐ and age‐driven adipogenic and osteogenic differentiation. Consequently, it provides an effective regulatory strategy for improving repair outcomes in patients with age‐related tendinopathies.

### 2.7 P/H@Lipo Reduces Inflammatory Levels in Young TDSCs and Regulates Macrophage Reprograming

To evaluate the effects of P/H@Lipo on inflammatory levels and mitochondrial function in young TDSCs, we employed DCFH‐DA and MitoSOX Red to detect intracellular and mitochondrial ROS levels, respectively. JC‐1 and Mito Tracker Red CMXRos were utilized to assess mitochondrial membrane potential, while the mPTP assay reagent was employed to evaluate the open state of the mitochondrial permeability transition pore.

**FIGURE 6:**
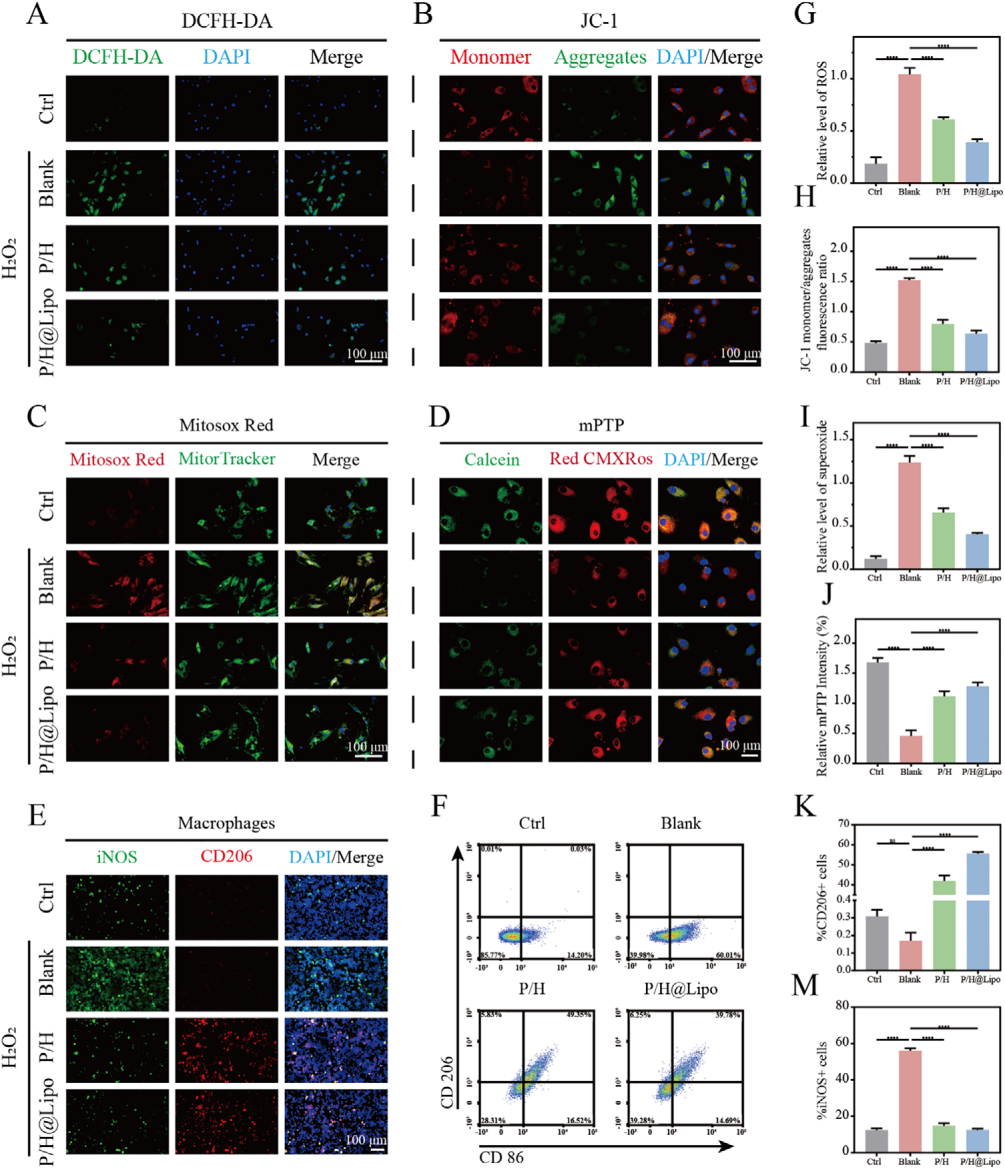
P/H@Lipo reduces inflammation levels in young TDSCs and regulates macrophage reprogramming. A) Images showing intracellular ROS levels detected with DCFH‐DA. B) Immunofluorescence staining images detecting mitochondrial membrane potential with JC‐1. Immunofluorescence staining images detecting mitochondrial permeability transition pores and membrane potential. C,D) Immunofluorescence images of mitochondrial permeability transition pores and membrane potential detected using Mitosox Red and Calcein AM indicators. Cell nuclei stained with DAPI. E,F) Immunofluorescence staining of iNOS and CD206 in macrophages; flow cytometric analysis of CD86 and CD206 in macrophages. G–J) Semi‐quantitative analysis of relative fluorescence intensity in (A)–(D). K–M) Percentage of iNOS+ cells and CD206+ cells (*n* = 3 per group). Data are expressed as mean ± SD (*p < 0.05; **p < 0.01; ***p < 0.001).

Cellular ROS probe detection revealed that HPSe significantly reduced H_2_O_2_‐induced ROS levels, with effects further enhanced following combined intervention with anti‐aging drugs (Figure 6A,G). Under H_2_O_2_ stimulation, the mitochondrial membrane potential of young TDSCs markedly increased, which was significantly restored following HPSe intervention. Co‐administration with anti‐aging drugs further stabilized the membrane potential, indicating that HPSe effectively maintains mitochondrial function, while anti‐aging drugs may enhance this effect by improving the overall microenvironment (Figure 6B,H). Mitochondrial superoxide staining results were consistent with this, showing HPSe significantly reduced superoxide levels, with the combined drug group also exhibiting a certain synergistic effect (Figure 6C,I). mPTP detection revealed that H_2_O_2_ caused substantial mPTP opening, which was significantly inhibited by HPSe treatment, with the P/H@Lipo complex system showing the most pronounced intervention effect (Figure 6D,J).

Regarding macrophages, ROS probes indicated that HPSe markedly reduced inflammatory levels within macrophages (Figure S9). Immunofluorescence staining revealed that H_2_O_2_ intervention promoted iNOS^+^ M1 macrophage polarization while inhibiting CD206^+^ M2 polarization; HPSe treatment markedly reduced M1 macrophage numbers while substantially increasing M2 macrophages, with no discernible difference observed following combined anti‐aging drug administration (Figure 6E,K–M). Flow cytometry further confirmed these finding. During co‐culture with aged TDSCs, M1 macrophages constituted approximately 14.2% of the population, while M2 macrophages were scarcely detected. Following H_2_O_2_ stimulation, the proportion of M1‐type macrophages increased to approximately 60%; however, HPSe intervention reduced the proportion of M1 macrophages to 16.8% and elevated M2 macrophages to approximately 4.75%, with combined anti‐aging drugs showing no significant alteration in this ratio (Figure 6F).

The above results fully demonstrate that the P/H@Lipo hydrogel system can effectively alleviate inflammatory responses in young TDSCs, improve mitochondrial function, and regulate the polarization of macrophages from pro‐inflammatory M1 to anti‐inflammatory M2 types. This facilitates a shift in the microenvironment from a pro‐inflammatory to an anti‐inflammatory state.

The functional impairment of young TDSCs is closely associated with excessive ROS in the microenvironment. These ROS disrupt mitochondrial structure and function, leading to impaired oxidative respiration. This manifests as excessive intracellular ROS accumulation, elevated mitochondrial membrane potential and superoxide levels, abnormal opening of the mPTP, and activation of Bax/Bak. These changes subsequently induce mtDNA leakage and trigger inflammatory apoptosis. Previous studies demonstrated that the P/H@Lipo hydrogel system significantly delays aging and apoptosis in young TDSCs. This research further indicates that the system effectively clears ROS, thereby reducing intracellular inflammatory levels, stabilizing the mitochondrial membrane potential, and inhibiting mPTP opening and Bax/Bak activation. This consequently reduces mtDNA leakage, which may represent a key mechanism underlying the previously observed attenuation of aging and apoptosis.

Macrophages play a pivotal role in regulating tendon inflammation. Prior to the onset of age‐related tendinopathy, a high proportion of senescent cells continuously secrete SASP factors, recruiting macrophages and stimulating their pro‐inflammatory M1 polarization. This induces a chronic low‐grade inflammatory state within the tissue, during which the microenvironment maintains relative homeostasis. Upon tendon injury, the inflammatory response rapidly escalates and becomes uncontrolled, disrupting microenvironmental homeostasis. Numerous macrophages are recruited and polarized toward the M1 phenotype, further exacerbating inflammation. This leads to increased apoptosis of TDSCs, forming a vicious cycle of “injury‐inflammation amplification‐apoptosis.” The persistently deteriorating inflammatory microenvironment prevents young TDSCs from effectively performing their reparative functions, constituting the key reason for the chronic, intractable nature of age‐related tendinopathy [35, 36].

This study demonstrates that the P/H@Lipo hydrogel system not only scavenges ROS but also regulates macrophage reprogramming, promoting their transition from M1 to M2 type. This shifts the inflammatory microenvironment from a proinflammatory to an anti‐inflammatory state, providing young TDSCs with a microenvironment conducive to repair. Ultimately, this facilitates tissue regeneration and functional recovery in tendinopathy.

### 2.8 Mechanistic Study of HPSe Regulation of Proliferation and Differentiation in Young TDSCs

**FIGURE 7:**
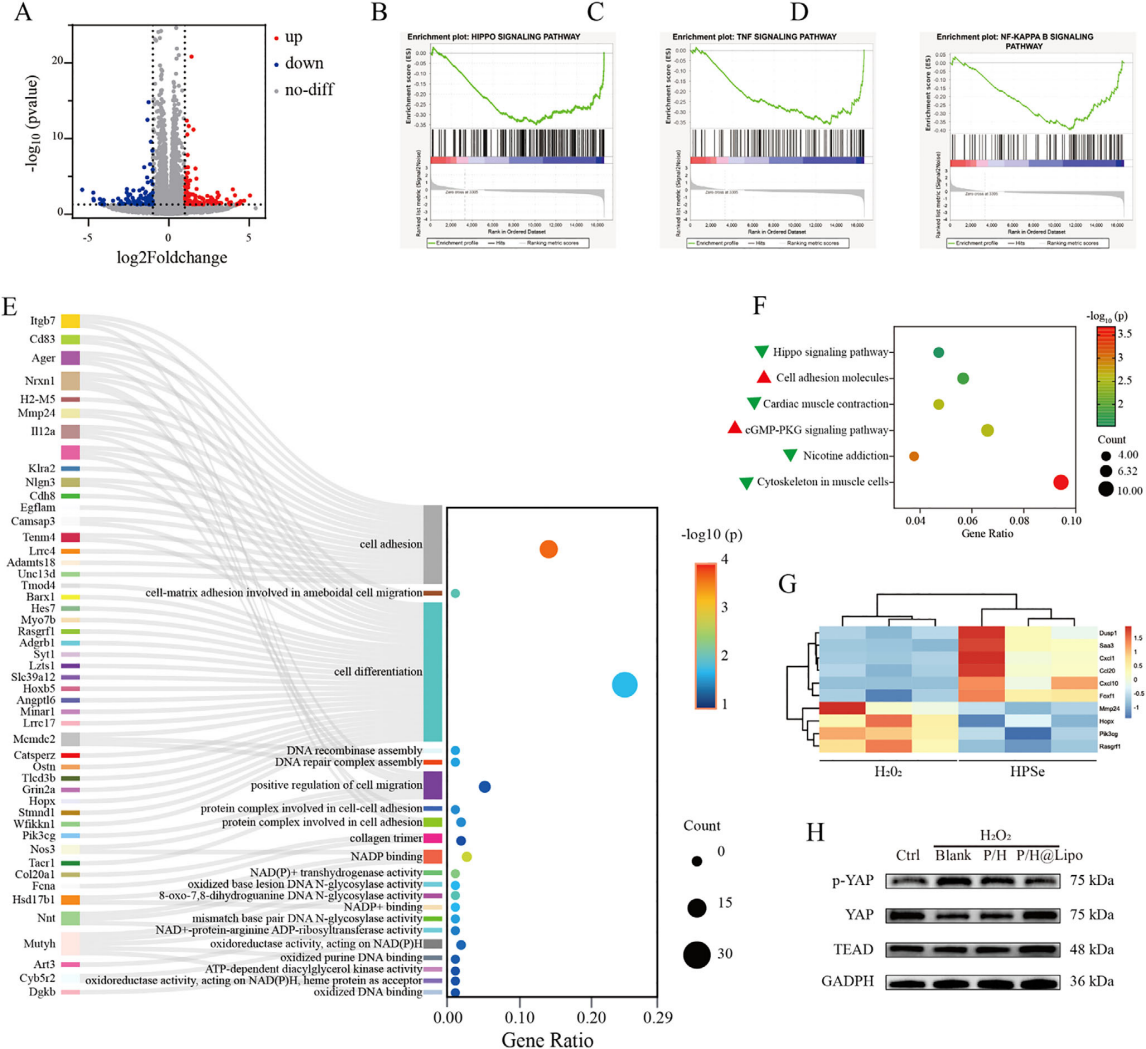
Mechanistic study of HPSe regulation of proliferation and differentiation in young TDSCs. A) Volcano plot of differentially expressed genes (gray: no significant difference; red: upregulated genes; blue: downregulated genes). B) GSEA analysis of the Hippo pathway. C) GSEA analysis of the TNF pathway. D) GSEA analysis of the NF‐κB pathway. E) Upregulation gene GO enrichment analysis combined with gene Sankey diagram. F) KEGG pathway enrichment analysis for significantly differentiated pathways (Red: upregulated genes; Green: downregulated genes. G) Heatmap of gene expression related to cell proliferation, differentiation, and migration. H) Western blot images showing levels of YAP, p‐YAP, and TEAD proteins in the Hippo signaling pathway (*n* = 3 per group). Data are expressed as mean ± SD (*p < 0.05; **p < 0.01; ***p < 0.001).

To further investigate the mechanism by which HPSe improves the function of young TDSCs, this study performed transcriptome sequencing and bioinformatics analysis on cells from the HPSe‐treated group and the control group. Sequencing results revealed that HPSe intervention induced significant changes in the expression of 234 genes, with 102 genes upregulated and 132 genes downregulated (Figure 7A). Gene set enrichment analysis (GSEA) indicated that the Hippo, TNF, and NF‐κB signaling pathways were markedly downregulated following HPSe intervention (Figure 7B–D). GO enrichment analysis of upregulated genes, combined with a Sankey diagram, revealed that differentially expressed genes were primarily concentrated in cell differentiation, DNA damage repair, and cell adhesion, consistent with the previously observed HPSe‐maintained cell proliferation, tendinogenic differentiation, and migration phenotypes (Figure 7E). KEGG pathway enrichment analysis showed that the Hippo signaling pathway was significantly downregulated following HPSe intervention (Figure 7F). Heatmaps of gene expression related to cell proliferation, differentiation, and migration analyzed similarities and differences in gene expression patterns (Figure 7G and S10D). Subsequent Western blot experiments further validated HPSe's inhibitory effect on key Hippo signaling pathway proteins, confirming the reliability of transcriptomic analysis results at the protein level (Figure 7G and S10A–C).

Young TDSCs play a crucial role in the repair process of age‐related tendinopathy. Preliminary experimental results indicate that HPSe effectively alleviates the inhibitory effects of ROS on the proliferation, migration, and tendinogenic differentiation of young TDSCs. To further elucidate its mechanism of action, transcriptome sequencing was performed along with in‐depth analysis of cells treated with HPSe. GSEA analysis revealed significant downregulation of the Hippo, NF‐κB, and TNF signaling pathways following HPSe intervention. The Hippo pathway regulates stem cell proliferation and differentiation [37], the NF‐κB pathway is closely associated with inflammatory responses [38], and the TNF pathway governs the process of apoptosis [39]. These findings suggest that HPSe may maintain the stemness of young TDSCs, mitigate inflammatory responses, and inhibit apoptosis by synergistically suppressing these three signaling pathways, consistent with the observed phenotypic improvements. GO enrichment analysis of the up‐regulated genes revealed significant overrepresentation in the Molecular Function (MF) category, primarily involving oxidized purine/mismatch base DNA N‐glycosylase activity, 8‐oxoguanine repair, NAD(P)H oxidoreductase activity, and DNA repair complex assembly. These findings indicate that cells enhance base excision repair and oxidative damage clearance to cope with an inflammatory microenvironment. Concurrently, terms such as “cell adhesion,” “cell–substrate adhesion,” and “positive regulation of cell migration” were enriched, implying that the up‐regulated genes also participate in extracellular matrix remodeling and increased cell motility. Furthermore, cellular component terms—including collagen trimer and cell adhesion protein complex—were elevated, further supporting activation of adhesion and matrix secretion functions. Collectively, the GO results reflect coordinated upregulation of DNA repair, anti‐oxidative stress, cell adhesion, and migration in HPSe‐treated TDSCs, aligning with our earlier observations. Functionally, this pattern may underlie HPSe‐mediated maintenance of TDSC stemness and is closely related to tendon stem cell injury repair or post‐inflammatory remodeling. In addition, Figure 7G presents a heatmap of proliferation‐, differentiation‐, and migration‐related genes; clustering analysis reveals both similarities and differences in expression patterns. Further analysis of differentially expressed genes revealed marked downregulation of several genes closely associated with the Hippo pathway, including Wnt8a, Ccn2, Ppp2r2b, Bmp5, and Gdf5. KEGG enrichment analysis also identified the Hippo signaling pathway among significantly altered pathways.

To validate the sequencing results, we performed Western blot analysis to detect key proteins in the Hippo‐YAP‐TEAD pathway. Our results demonstrated reduced levels of phosphorylated YAP following HPSe intervention, indicating suppression of the Hippo signaling pathway. This consequently allowed increased unphosphorylated YAP to enter the nucleus, thereby enhancing TEAD‐mediated transcriptional activity. This mechanism may represent a crucial molecular basis for HPSe in maintaining the stemness and self‐renewal capacity of young TDSCs (Figure S10D).

This study utilized transcriptomic analysis and experimental validation to demonstrate that HPSe mitigates inflammation and apoptosis by inhibiting the Hippo, NF‐κB, and TNF signaling pathways. This promotes the proliferation and tendinogenic differentiation of young TDSCs, thereby providing novel mechanisms and therapeutic strategies for treating age‐related tendinopathies.

### 2.9 P/H@Lipo Promotes the Repair of Age‐Related Tendinopathy within the Body

**FIGURE 8:**
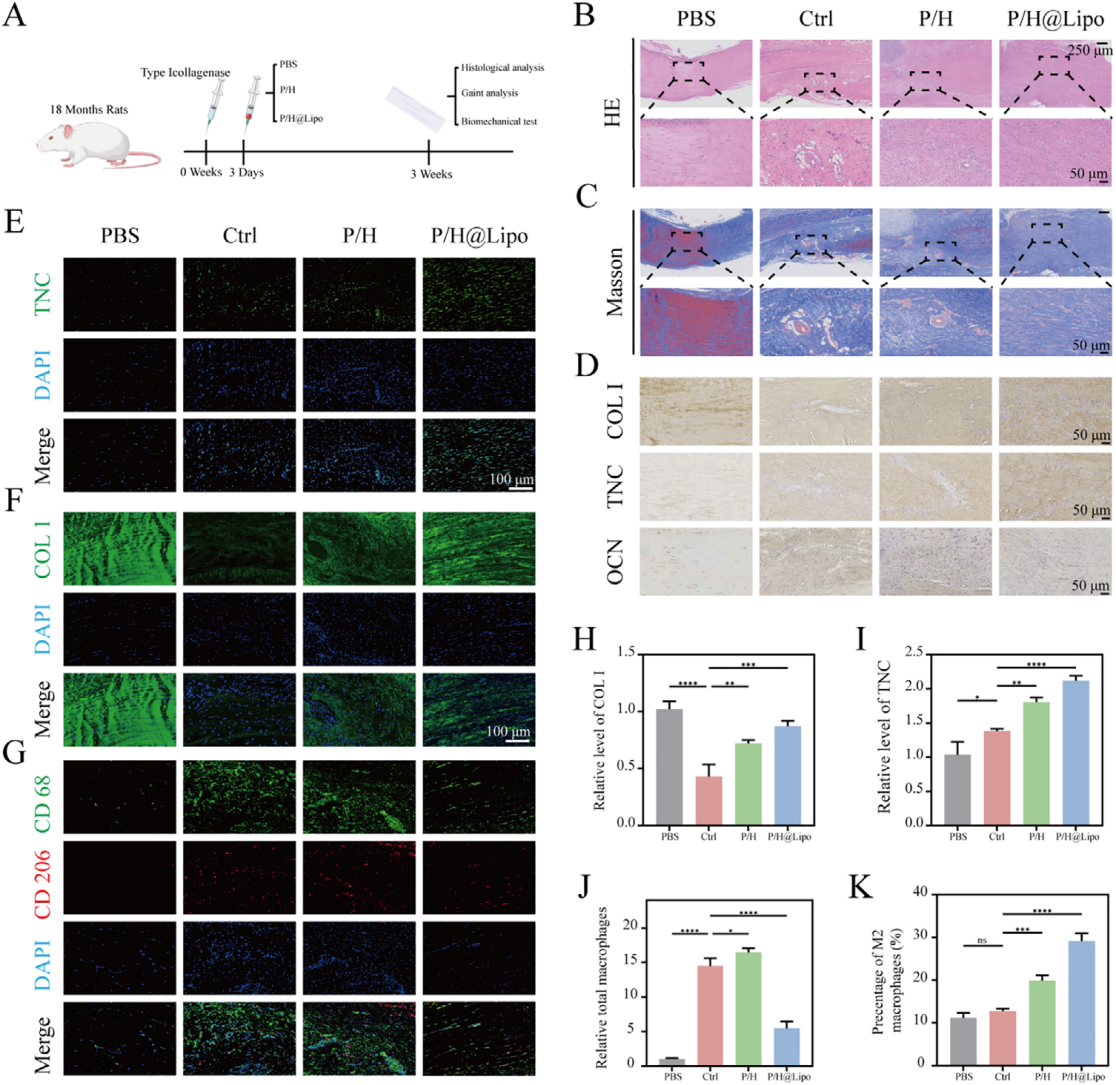
Histological assessment of P/H@Lipo‐mediated repair of senile tendinopathy in vivo. A) Schematic diagram of rat model establishment and subsequent procedures for senile tendinopathy. B–D) H&E staining, Masson staining, and immunohistochemical staining images for COL I, TNC, and OCN in tendons from different groups at 3 weeks post‐injury. E,F) Immunofluorescence images of COL I and TNC in tendons from different groups at 3 weeks post‐injury. G) Dual immunofluorescence staining for CD68 and CD206 in tendons from different groups at 3 weeks post‐injury. H–K) Relevant semi‐quantitative analysis (*n* = 3 per group). Data are expressed as mean ± SD (*p < 0.05; **p < 0.01; ***p < 0.001).

To evaluate the in vivo therapeutic efficacy of P/H@Lipo for age‐related tendinopathy, this study employed 18‐month‐old aged rats. An animal model was established via local injection of type I collagenase. Three days post‐modelling, rats were randomly assigned to receive local injections of the respective formulations. Prior to the formal experiment, we conducted an exploratory preliminary study comparing tendon repair status at weeks 3, 4, and 5 post‐injury. By Week 3, the repair quality (collagen alignment and maturity) in the P/H@Lipo group had significantly surpassed that of the P/H group, while the P/H group also markedly outperformed the repair‐stalled Ctrl group (Figure 8B,C and S11A,B). This advantage stems from the synergistic anti‐aging effects of the STING inhibitor (H‐151) delivered by P/H@Lipo targeting HPSe, which can earlier interrupt the vicious cycle of “inflammation‐aging.” By Week 4, the P/H@Lipo group had entered a repair plateau phase (with abundant mature collagen), while the P/H group remained lagging. By Week 5, the final repair quality of both the P/H and P/H@Lipo groups converged, though P/H@Lipo achieved faster repair. These findings indicate that: P/H@Lipo accelerates high‐quality repair through anti‐aging mechanisms; The most significant differences between treatment groups were observed at 3 weeks, demonstrating the clinical advantage of “accelerated healing” (i.e., patients may regain function sooner). Prolonging the observation period diluted this core advantage, and differences between treatment groups became negligible beyond 5 weeks. Therefore, week 3 was selected as the observation time point for footprint analysis, macroscopic Achilles tendon examination, and histological assessment in animal experiments (Figure 8A).

Footprint analysis revealed that rats in the PBS group exhibited larger footprint areas, whereas those in the collagenase‐induced model group (Ctrl) demonstrated markedly shallower footprints due to Achilles tendon pain and functional impairment. Footprint depth partially recovered in the HPSe‐treated group, while the P/H@Lipo‐treated group exhibited significantly increased footprint depth approaching normal levels, indicating pronounced therapeutic efficacy of this system (Figure S13A). Macroscopic examination after 3 weeks of treatment revealed that the Achilles tendon in the PBS group appeared white, with a clear structure and a smooth surface. In contrast, the Ctrl group exhibited a pale‐yellow tendon with marked edema and thickening, severely adhered to surrounding tissues. The HPSe group showed reduced edema and adhesion. The P/H@Lipo group demonstrated near‐complete recovery of tendon morphology, appearing white with intact structure (Figure S13B).

H&E staining revealed that the PBS group exhibited a low cell count with regular, orderly arrangement and no vascular proliferation. The Ctrl group demonstrated extensive vascular proliferation, disorganized cell arrangement, and marked proliferation. The HPSe group showed reduced vascular and cellular proliferation with a relatively regular arrangement. The P/H@Lipo group exhibited almost no vascular proliferation, relatively well‐organized cell arrangement, and significantly reduced cellular proliferation compared to the Ctrl group (Figure 8B). Masson staining revealed that the PBS group predominantly contained mature red collagen; the Ctrl group exhibited only minor amounts of blue new collagen; the HPSe group showed increased blue collagen; and the P/H@Lipo group displayed substantial coexistence of red mature collagen and blue newly synthesized collagen, indicating optimal repair efficacy (Figure 8C). Protein expression levels of COL I and TNC reflect tendon tissue repair status. Immunohistochemical staining revealed high expression of COL I and TNC in the PBS group; significantly downregulated expression in both proteins in the Ctrl group; slight recovery in the HPSe group; and marked restoration in the P/H@Lipo group (Figure 8D and S12A,B). The expression level of osteocalcin (OCN) reflects the tendency toward ossification following tendon injury. Results showed that compared with normal tendons (Ctrl group), OCN expression was significantly elevated in the model group (Blank group), indicating activation of heterotopic ossification after tendon injury. Following P/H treatment, OCN expression was downregulated (Figure 8D and S12A,B). The combined P/H@Lipo therapy further significantly suppressed OCN expression, restoring it to near‐normal levels. These findings indicate that P/H@Lipo effectively antagonizes injury‐induced abnormal osteogenic differentiation, thereby preventing secondary heterotopic ossification in age‐related tendinopathy in vivo. Immunofluorescence staining further confirmed that the PBS group exhibited regular cellular arrangement, abundant COL I, and low TNC expression; the Ctrl group showed disorganized cells, marked inflammatory infiltration, degraded COL I, and elevated TNC; the P/H@Lipo group demonstrated significantly increased COL I secretion and further upregulation of TNC, indicating favorable repair progression (Figure 8E,F). Dual immunofluorescence staining for CD68 and CD206 revealed that P/H@Lipo reduced CD68+ macrophage infiltration while promoting CD206+ macrophage polarization (Figure 8G). This aligns with in vitro findings, demonstrating P/H@Lipo's anti‐inflammatory effects by mitigating macrophage infiltration and facilitating macrophage reprogramming.

Biomechanical performance testing demonstrated significant improvements in the failure stress, Young's modulus, and stiffness of tendons in the P/H@Lipo group (Figure S13C–F). Furthermore, no notable pathological alterations were observed in major organ sections, indicating favorable biosafety of this system (Figure S13G).

This study comprehensively evaluated the in vivo therapeutic efficacy of the P/H@Lipo system using a collagenase‐induced tendinopathy model in aged rats. Functional, histological, and protein expression analyses consistently demonstrated that P/H@Lipo significantly promoted tendon structural and functional recovery. Its reparative mechanism likely involves multiple pathways: first, alleviating oxidative stress by scavenging ROS, thereby suppressing inflammatory responses and abnormal cellular differentiation; second, modulating macrophage polarization to promote macrophage reprogramming and improve the local immune microenvironment, thus creating favorable conditions for tendon regeneration.

It is noteworthy that P/H@Lipo treatment not only promotes collagen synthesis and maturation but also significantly enhances the biomechanical properties of tendons, indicating that repaired tendons exhibit sound structural integrity and mechanical function. Concurrently, this system demonstrates favorable biosafety, providing experimental evidence for its further clinical translation.

Overall, P/H@Lipo effectively promotes the repair of age‐related tendinopathy through multiple synergistic mechanisms, including antioxidant effects, macrophage reprogramming, and regeneration promotion, thereby offering a promising therapeutic strategy for age‐related tendon disorders.

## 3 Conclusion

This study compared the functional differences between aging and young TDSCs, revealing a marked decline in the proliferation and differentiation capacity of aging TDSCs. Given the coexisting inflammatory and senescent characteristics within the aging tendon microenvironment, we developed P/H@Lipo, a ROS‐responsive dual‐targeting hydrogel system, as a core anti‐inflammatory and anti‐aging strategy. This system synergistically regulates mitochondrial‐nuclear communication to treat age‐related tendinopathies. Following local injection, P/H@Lipo responds to elevated ROS levels at the lesion site via its borate ester bonds, enabling on‐demand release of its loaded components: the STING inhibitor H‐151‐targeting liposome (L‐Lipo@H‐151) and the selenium nanocatalyst HPSe. Specifically, L‐Lipo@H‐151 employs a type I collagen‐targeting peptide for precise delivery of H‐151 to TDSCs, inhibiting the cGAS‐STING signaling pathway to counteract cellular senescence. HPSe targets TDSCs via HA‐mediated recognition, scavenging ROS to preserve mitochondrial structure and function. This reduces mtDNA leakage and downstream cGAMP production, synergistically inhibiting STING pathway activation with H‐151. By regulating mitochondrial‐nuclear communication, the system effectively delays aging in young TDSCs and maintains their tendinogenic potential. Mechanistic studies indicate that HPSe further enhances TDSC stemness and self‐renewal capacity by inhibiting the Hippo signaling pathway. In addition, P/H@Lipo induces macrophage reprogramming (M1→M2), promoting a shift in the local tendon immune microenvironment from pro‐inflammatory to anti‐inflammatory states, thereby establishing a favorable regenerative microenvironment for TDSC function. This study proposes a novel “targeted delivery‐multi‐pathway synergistic” therapeutic strategy for age‐related tendinopathy. However, while this study demonstrates P/H@Lipo's potent capacity to promote senescent tendinopathy repair through cellular and animal experiments, considering clinical translation, subsequent experimental studies will employ fluorescently labeled or radioactively labeled tracing techniques to systematically investigate P/H@Lipo's distribution, release kinetics, and metabolic status within animal models.

## 4 Experimental Section

### 4.1 Isolation, Characterization, and In Vitro Model of Rat Tendon Stem Cells

After euthanizing the rats, their Achilles tendons were isolated and removed, after which they were rinsed three times with PBS. Using ophthalmic scissors, excess connective tissue such as synovium, blood vessels, and muscles was removed from the tendon surface. The samples were then rinsed once more using PBS, then rinsed twice with a penicillin‐streptomycin solution. Finally, they were rinsed twice using PBS. The knee joints of the rats were disinfected in a sterile environment. Using ophthalmic scissors, the tendon tissues were then cut into fragments approximately 2 mm long. After rinsing once with PBS, the fragments were collected into a 50‐mL centrifuge tube. Trypsin was added for 10 min to facilitate digestion, after which PBS was added to terminate digestion before being centrifuged to collect the pellet. For every 100 mg of tendon tissue, 2 mL of type I collagenase (3 mg/mL) was added and incubated at 37 °C in a 5% CO_2_ cell culture incubator for 24 h. Digestion was terminated using an equal volume of F‐12 medium containing 10% fetal bovine serum before being centrifuged again. The supernatant was removed, the pellets were resuspended in complete medium, and then uniformly seeded into cell culture flasks for cultivation. The cell status was monitored regularly, and the medium was changed every 2 days. Cells were then passaged when confluence reached 80%–90% in flasks. The primary cells were designated as P0 and subsequent cells were designated as P3–P5 passages. When the cells reached 90% confluence and adhered to surfaces, they were viewed using a vertical fluorescence microscope (Olympus DP74, Olympus Corporation, Tokyo, Japan). In addition, immunofluorescent staining with COL1, SCX, MKX, and TNMD proteins was performed, followed by confocal fluorescence microscopy imaging. Cell surface antibody detection using CD90, CD44, CD106, and CD34 was conducted via flow cytometry (Agilent).

To simulate the microenvironment of aging tendinopathy where young TDSCs reside, P3‐passaged TDSCs were co‐cultured with P12‐passaged TDSCs as the Ctrl group. For oxidative stress simulation, 100 µmol of H_2_O_2_ was added to the Ctrl group. The P/H group involved co‐culturing with hydrogels formed solely from PVA and HPSe supplemented with 100 µmol of H_2_O_2_. The P/H@Lipo group involved co‐culture with a hydrogel composed of PVA, HPSe, L‐Lipo@H‐151, and 100 µmol of H_2_O_2_.

### 4.2 Synthesis and Characterization of HPSe

HA (0.25 mm) and PBA (0.25 mm) were dissolved and mixed, followed by the addition of DMTMM (0.25 mm). The mixture was stirred at 22 ± 1°C for 72 h, then placed in a 6–8 kDa dialysis bag. After 4 days of dialysis, the mixture was freeze‐dried to obtain the HA‐PBA solid. Vitamin C and sodium selenite were then mixed at a 1:4 ratio and stirred at 22 ± 1 °C for 1 h. A pre‐prepared 1.5 mg/mL HA‐PBA solution was subsequently added and stirred at room temperature for 6 h. The solution turned orange‐red, followed by dialysis in a 300 kDa dialysis bag for 48 h. Freeze‐drying yielded the HPSe solid, which was then labeled with coumarin 6 (C6) to yield C6@HPSe. The synthesis method was identical to the above. Prior to initiating the redox reaction, 250 µL of a 1 mg/mL C6 solution (dissolved in DMSO) was added to the reaction system.

HA‐PBA was characterized using ultraviolet/visible/near‐infrared spectroscopy (UV/vis/NIR) and liquid nuclear magnetic resonance (NMR) (Germany‐Bruker‐AVANCE III HD 600), and the grafting rate of PBA was calculated. The morphology of HPSe was characterized using transmission electron microscopy (TEM, Thermo Fisher Scientific Talos F200S, USA) at an acceleration voltage of 120 kV. The microstructure and composition of HA‐SeNPs were examined by combining TEM with energy‐dispersive X‐ray spectroscopy (EDS). Surface chemical composition of HPSe was analyzed using X‐ray photoelectron spectroscopy (XPS, USA‐Thermo Fisher‐ESCALAB Xi+). The crystal structure of HPSe was analyzed using XRD and compared with the standard Se pattern. The zeta potential and particle size distribution of HPSe were determined using DLS (DLS, Zetasizer Nano ZS, Malvern Panalytical, Worcestershire, UK). To validate the colloidal stability of HPSe, freshly synthesized HPSe was completely dissolved in PBS containing 10% goat serum (pH = 7.4) and placed on a constant‐temperature shaking incubator at 37°C. Particle size measurements were performed daily on 200 µL of the HPSe solution over a 12‐day period. To investigate the optimal concentration of HPSe, H_2_O_2_ scavenging assays and CCK‐8 cell proliferation and cytotoxicity assays were performed. Five HPSe concentration groups were established: 50, 100, 150, 200, and 250 µg/mL. Each group was dissolved in 1 mm H_2_O_2_ solution. After 10 min, residual H_2_O_2_ content was measured to evaluate HPSe's scavenging efficacy. Five different HPSe concentration groups were established: 50, 100, 150, 200, and 250 µg/mL. Co‐culture these groups with TDSCs and measure absorbance at 450 nm to evaluate cell viability.

### 4.3 Preparation and Characterization of L‐Lipo@H‐151

Egg yolk lecithin, cholesterol, DSPE‐PEG2000, DSPE‐PEG2000‐NHS, DSPE‐PEG2K‐Mal‐CLHERHLNNN (30:6:6:1:1, w/w), and H‐151 were weighed. All components were dissolved in chloroform and transferred to a round‐bottom flask. The chloroform was evaporated using a rotary evaporator (40°C, 2 h) until it was dry, forming a thin film on the flask. PBS was added to rehydrate the mixture, yielding a liposome suspension. Using a probe ultrasonicator, sonication was performed at 5% power (2 s on and 2 s off, for a total of 5 min). The liposomes were then passed through a 200 nm polycarbonate membrane by repeated squeezing (10 cycles), yielding H‐151‐loaded grafted targeting peptide liposomes (L‐Lipo@H‐151). L‐Lipo@H‐151 was labeled with coumarin 6 (C6) to yield C6@L‐Lipo@H‐151. The synthesis method was identical to the above. Prior to rotary evaporation, 250 µL of a 1 mg/mL C6 solution (dissolved in DMSO) was added to the reaction system.

The morphology of HPSe was characterized using TEM (Thermo Fisher Scientific Talos F200S, USA). The zeta potential and particle size distribution of L‐Lipo@H‐151 were determined by DLS (Zetasizer Nano ZS, Malvern Panalytical, Worcestershire, UK). To validate the stability of L‐Lipo@H‐151, freshly synthesized L‐Lipo@H‐151 was dissolved in PBS (pH = 7.4) supplemented with 10% goat serum and incubated on a 37°C shaking incubator. Particle size measurements were performed at 1, 12, 24, 36, 48, 60, and 72 h. To determine the drug loading capacity and encapsulation efficiency of L‐Lipo@H‐151, 100 µL of L‐Lipo@H‐151 was dissolved in 900 µL of methanol. After 30 s of ultrasonic treatment, the mixture was centrifuged at 12,000 rpm for 10 min. The concentration of H‐151 was measured using a UV spectrophotometer, and the content was calculated as follows:

DL%=Wd/Ws×100%;EE%=Wd/Wt×100%

(*W*_d_ represents the total mass of H‐151 in L‐Lipo@H‐151, *W*_s_ denotes the total mass of L‐Lipo@H‐151, and *W*_t_ indicates the initial mass of H‐151 added during L‐Lipo@H‐151 preparation.)

To verify H‐151 release from the liposomes, 1 mL of Lipo@H‐151 and L‐Lipo@H‐151 were loaded into a dialysis bag (molecular weight cut‐off: 2000 Da). The bag was immersed in 50 mL of PBS (pH = 7.4) containing 1% Tween‐80 and stirred at 50 rpm at 37°C. At different time points (0, 6, 12, 18, 24, 30, 36, 42, and 48 h), 0.1 mL of the dialysate was sampled, and an equal volume of dialysate was added. Drug concentration was measured using a UV spectrophotometer. For the targeting assessment of L‐Lipo@H‐151 to TDSCs, fluorescently labeled liposomes prepared as described above were co‐cultured with TDSCs. After 2 days, flow cytometry analysis and confocal fluorescence microscopy observations were performed, and the images were captured.

### 4.4 Preparation and Characterization of PVA/HPSe@Lipo Hydrogels

Pre‐configured solutions containing 2.5% PVA by mass fraction, 200 µg/mL of HPSe, and L‐Lipo@H‐151 were directly mixed. After stirring with a glass rod for 10 s, gelation occurred. The mixture was freeze‐dried for subsequent experiments. Verification of injectability for the PVA/HPSe@Lipo hydrogel composite system was performed by loading the hydrogel into a 5‐mL syringe and directly injecting it through the needle into PBS solution. Morphological observation of the freeze‐dried hydrogel was conducted using SEM (Germany‐ZEISS‐GeminiSEM 360). Dynamic rheological testing of the hydrogel was conducted at 22 ± 1°C using a rheometer (Germany‐NETZSCH‐Kinexus Prime lab+). FTIR spectroscopy (Japan‐SHIMADZU‐IRTracer‐100) was employed to characterize both PVA and the PVA/HPSe@Lipo hydrogel composite system, confirming the presence of borate ester bonds. The PVA/HPSe@Lipo hydrogel composite system was sequentially immersed in PBS solution and H_2_O_2_ solution, placed in a 37 °C incubator, and shaken at 80 rpm. Every 12 h, 100 µL of supernatant was collected and mixed with an equal volume of dialysis buffer. HPSe concentration was measured using a UV spectrophotometer to calculate the HPSe release ratio. After co‐culturing PVA/HPSe, PVA/HPSe@Lipo with TDSCs, and RAW 264.7 cells for 3 days, gel toxicity was assessed using a cell viability/apoptosis assay kit, including a blank control. TDSCs and RAW 264.7 cells were divided into two groups each. One group was cultured with PBS solution, and the other with HA solution (1 mg/mL) for 30 min. Subsequently, PVA/HPSe@Lipo was co‐cultured with TDSCs and RAW 264.7 cells. Flow cytometry analysis and confocal fluorescence microscopy were performed at 0, 2, 4, and 8 h to validate cellular targeting.

### 4.5 Using Immunofluorescence Staining for Protein Localization and Expression Analysis

After treating different groups of TDSCs for 72 h, they were washed twice with PBS and fixed with 4% PFA. After blocking with 1% BSA (Servicebio, China), the cells were washed three more times with PBS. Primary antibodies targeting COL I, TNMD, MKX, SCX, P16, and P21 (Proteintech Group, Inc., China) were added, and the cells were incubated overnight at 4°C. After primary antibody incubation, cells were washed three times with PBS on a rocking platform. They were then incubated for 60 min at room temperature with either CY3‐conjugated goat anti‐rabbit secondary antibody or FITC‐conjugated goat anti‐rabbit secondary antibody (Affinity Biosciences, USA). Following secondary antibody incubation, the cells were stained with FITC‐ or rhodamine‐labeled phalloidin for 30 min to visualize their cytoskeleton. The cells were washed three times with PBS and stained with DAPI for 10 min to visualize their nuclei. Cells were observed and photographed using confocal fluorescence microscopy. Fluorescence intensity was quantified using ImageJ software (version 1.54) to evaluate the effects of different interventions on key protein expression and subcellular localization in TDSC cells.

### 4.6 Assessment of Inflammation and Apoptosis Levels in TDSCs via Confocal Microscopy and Flow Cytometry

ROS and superoxides were detected using the ROS Assay Kit (Beyotime, China) and MitoSox Mitochondrial Superoxide Indicator (Thermo Scientific, USA) according to the manufacturers’ instructions. Mitochondrial membrane potential was detected using JC‐1 (Beyotime, China) and Mito‐Tracker Green (Beyotime, China). Detection of mPTP opening was performed using the mPTP Detection Kit (Beyotime, China) and Red CMxRos (Beyotime, China). Briefly, after treating TDSCs with different groups for 72 h, the probes were mixed with serum‐free medium and diluted to working concentrations. After 20 min of incubation, the nuclei were stained with DAPI. Suitable images were then captured using confocal fluorescence microscopy. Fluorescence intensity was quantified using ImageJ software (version 1.54, National Institutes of Health, Bethesda, MD, USA) to assess the effects of different interventions on inflammatory levels in TDSCs.

DNA damage and apoptosis in TDSCs were studied using immunofluorescence staining with γ‐H2AX and TUNEL (Beyotime, China) according to the kit instructions (Cell Signaling Technology, USA). Cell apoptosis was detected using the Annexin V‐FITC/PI Apoptosis Kit (Servicebio, China) according to the manufacturers’ protocol. Briefly, after treating TDSCs with different groups for 72 h, cells were fixed and permeabilized, then incubated with γ‐H2AX or TUNEL reaction buffer for 60 min. Images were subsequently captured using confocal fluorescence microscopy. Flow cytometric analysis of apoptosis was performed as follows: After 72 h of treating the different groups, TDSCs were gently washed with PBS and incubated in the dark for 20 min with Annexin V‐FITC and propidium iodide (PI) staining solutions. Samples were then immediately analyzed using flow cytometry (Agilent, China).

### 4.7 Protein Blotting Analysis

Cells were lysed using RIPA lysis buffer (Biyun Tian, China) containing protease and phosphatase inhibitors, followed by centrifugation to collect the supernatant. The respective protein concentrations were determined using the BCA Protein Assay Kit (Yasei, China). Equal volumes of protein samples were loaded onto a 12% SDS‐PAGE gel (Yasei, China) for electrophoresis. After electrophoresis, the gel was transferred to a PVDF membrane (Millipore, USA). The PVDF membrane was blocked with Protein‐Free Rapid Blocking Solution (Yasei, China) for 10 min, then incubated overnight at 4°C with primary antibodies against COL I, TNMD, MKX, SCX, P16, P21, GAPDH, STING, p‐TBK1, TBK1, p‐IRF, IRF, p‐P65, P65, Bax, and Bcl‐2 at 4°C overnight (Wuhan Sanying, China). After washing with TBST buffer, membranes were incubated with secondary antibodies (BODO, China) at 22 ± 1°C for 2 h, followed by additional washing with TBST. Chemiluminescent detection was performed using a developed solution (1:1) prepared according to the manufacturer's instructions (Biyun Tian, China). The membrane was exposed to the developing solution, and protein bands were visualized using a chemiluminescent imaging system (Genesys, USA). Each experiment was performed in triplicate. Protein expression was quantified using ImageJ software (version 1.54 National Institutes of Health, Bethesda, MD, USA).

### 4.8 Assessment of Tendon Maturity Levels in TDSCs

Plate Cloning Assay: To evaluate the self‐cloning capacity of TDSCs, cells were digested with 0.25% trypsin (Beyotime, China) and uniformly seeded at 200 cells per well in a 6‐well plate. Different treatments were applied, with medium changes every 3 days. After 12 days, cells were fixed with 4% PFA. One milliliter of crystal violet staining solution (Beyotime, China) was added to each well, stained for 10–20 min, then observed and photographed under a microscope. CFU counts were assessed using visual inspection and colony enumeration.

*Scratch Assay*: The scratch assay is employed to assess cell migration capacity. Specifically, TDSC cells are uniformly seeded into a six‐well plate. Once the cells have fully confluently covered the plate, a linear cell‐free zone is created by scratching the monolayer with the tip of a 200 µL pipette. After washing with PBS, photographs are taken by microscope. Subsequently, different treatment groups were applied. Photographs were taken again after 24 h, and the cell migration rate was calculated.

*Osteogenic Differentiation*: TDSCs were seeded into six‐well plates and cultured until 70% confluence was reached, after which different group interventions were applied. Cells were then induced to differentiate into osteoblasts using an osteogenic induction medium. The medium was changed periodically throughout the induction process. On day 21 of culture, the cells were stained using alizarin red solution (Beyotime, China).

*Adipogenic Differentiation*: TDSCs were seeded into 6‐well plates and cultured until 70% confluence was achieved, after which different interventions were applied to each group. The cells were then induced to differentiate into adipocytes using adipogenic induction medium. The medium was changed periodically throughout the induction process. On day 21 of culture, cells were stained with Oil Red O solution (Beyotime, China).

*Senescence‐Associated β‐galactosidase Staining*: SA‐β‐gal staining was performed using the β‐galactosidase staining kit (Beyotime, China) to assess the degree of cellular senescence. After treating TDSCs with different groups for 72 h, the cells were washed with PBS. A total of 1 mL of β‐galactosidase staining fixative was added to each well, and the cells were fixed at room temperature for 15 min. Following fixation, the fixative was aspirated, and the cells were washed three times with PBS for 3 min each. Afterward, the PBS was removed, and 1 mL of staining working solution was added to each well. The cells were incubated overnight at 37°C in a CO_2_ incubator. The cells were viewed and photographed under an optical microscope the following day. Senescent cells exhibit blue or blue‐green cytoplasm. The blue‐stained areas were measured using ImageJ software, and the percentage of positive cells was calculated to quantify the degree of cellular senescence.

### 4.9 Evaluation of Mitochondrial Structure and Function in TDSC Cell Lines

Upon stimulation with ROS in the inflammatory microenvironment, mtDNA leaks into the cytoplasm through the mPTP and BAK/BAX pores on the mitochondrial membrane. This mtDNA is then catalyzed by cGAS to produce cGAMP, which activates STING, ultimately triggering cellular senescence and apoptosis. mtDNA leakage was detected using immunofluorescence staining and qPCR. ATP levels were measured using an ATP assay kit (Abbkine China) to assess mitochondrial function. Mitochondrial structure was observed and evaluated using bio‐projection electron microscopy (TEM, Thermo Fisher Scientific Talos F200S, USA).

*dsDNA Immunofluorescence Staining*: After treating TDSCs with different groups for 72 h, they were washed twice with PBS and fixed with 4% PFA; blocking with 1% BSA (Servicebio, China) was performed before being washed three more times with PBS. The primary antibody (dsDNA specific, Proteintech, China) was added and incubated with the cells overnight at 4°C. After incubation with the primary antibody, the cells were washed three times using PBS on a rocking platform, followed by incubation with FITC‐conjugated goat anti‐rabbit secondary antibody (Affinity Biosciences, China) for 60 min at room temperature. After secondary antibody incubation, the cytoskeleton was stained using FITC‐ or rhodamine‐labeled phalloidin for 30 min. The cells were then washed three times using PBS, and the nuclei were stained with DAPI dye for 10 min. The cells were observed and samples photographed under confocal fluorescence microscope. Fluorescence intensity was quantified using ImageJ software (version 1.54, National Institutes of Health, Bethesda, MD, USA).

*Extraction of Cytoplasmic mtDNA and Quantitative qPCR Analysis*: mtDNA leakage was assessed by comparing cytoplasmic mtDNA with total DNA. After treating TDSCs with different groups for 72 h, cells were lysed in 1% NP‐40 buffer, incubated on ice for 15 min, then divided into two equal fractions. One fraction served as the source for total DNA, while the other was centrifuged at 13,000× *g* at 4°C for 15 min, with the supernatant collected as the cytoplasmic fraction. Cytoplasmic and total DNA samples were then isolated using the DNAeasy Blood & Tissue Kit (Qiagen Germany). qPCR was used to amplify the mtDNA‐specific gene ND1 (NADH dehydrogenase subunit 1) and the total DNA‐specific gene 18S rDNA. Primer sequences are provided in Table S1. Relative cytoplasmic mtDNA levels were calculated and plotted by normalizing cytoplasmic mtDNA Ct values against total DNA Ct values.

*ATP Content Detection*: ATP levels within cells treated with different groups were measured using an ATP detection kit (Abbkine, China). The experimental procedure was as follows: cells were thoroughly disrupted by sonication, heated at 100°C for 5 min, then centrifuged at 8,000 × *g* for 15 min at 4°C. The supernatant was collected for analysis. Subsequently, reagents were added as specified, incubated at 37°C for 20 min, and its absorbance was measured at 700 nm. ΔA_(test) was then calculated using the following equation:

A_(test) = A_(test) – A_(control) and ΔA_(standard) = A_(standard) – A_(blank).

The ATP concentration in each sample was also determined using the following formula:

ATP content (µmol/mL) = [CStandard × ΔA test ÷ ΔA standard × V1] ÷ (V3 × V1 ÷ V2) = 20 × ΔA test ÷ ΔA standard

*Observation of Mitochondrial Morphology by TEM*: After treating cells in different groups, they were fixed with 2.5% glutaraldehyde at 4°C for 12 h, followed by fixation with 1% osmium tetroxide at 4°C for 4 h. Following fixation, the cells were washed with PBS and dehydrated in a gradient of ethanol solutions (30%, 50%, 70%, 90%, and 100%) and 100% acetone for 20 min. Following dehydration, the samples were embedded in epoxy resin, sectioned into ultrathin slices, double‐stained with uranium acetate and lead citrate, and examined and photographed under a transmission electron microscope.

### 4.10 Transcriptome Sequencing and Analysis

In the transcriptome sequencing experiment, two groups were established, the H_2_O_2_ group and the PVA/HPSe group. After 3 days of respective interventions, total RNA was isolated and purified from each group using Trizol (Beyotime Biotechnology Co., Shanghai, China). The extracted RNA was stored at −80°C. Subsequently, the RNA samples were sent to Bioprofile in Shanghai, China, for RNA transcriptome sequencing.

### 4.11 Evaluation of Macrophage Reprogramming

Detect ROS levels in macrophages using the ROS Assay Kit (Beyotime, China) according to the manual. Briefly, after treating macrophages with different groups for 72 h, the probe was mixed with a serum‐free medium and diluted to the working concentration. After 20 min of incubation, the cell nuclei were stained using DAPI. Images were then captured using confocal fluorescence microscopy. Fluorescence intensity was quantified using ImageJ software (version 1.54, National Institutes of Health, Bethesda, MD, USA).

Macrophages were analyzed for polarization using dual immunofluorescence staining for CD206 and iNOS, and flow cytometry for CD86 and CD206. Specifically, macrophages were treated with different groups for 72 h, then fixed with 4% PFA. After blocking with 1% BSA (Servicebio, China), the cells were washed three times using PBS. Primary antibodies targeting CD206 and iNOS (Abcam, USA) were added, and cells were incubated overnight at 4°C. After primary antibody incubation, the cells were washed three times with PBS on a rocking platform. They were then incubated at room temperature for 60 min with secondary antibodies conjugated to CY3 or FITC (Affinity Biosciences, China). Following secondary antibody incubation, cells were washed three times with PBS and stained with DAPI for 10 min to visualize nuclei. Observation and imaging were performed using confocal fluorescence microscopy. Fluorescence intensity was quantified using ImageJ software (version 1.54, National Institutes of Health, Bethesda, MD, USA). After 72 h of treatment, macrophage surface markers CD206 and CD86 were analyzed by flow cytometry (Elabscience, China), analyzed on an Agilent flow cytometer.

### 4.12 Establishment and Validation of an In Vivo Animal Model for Senile Tendinopathy

Ninety healthy, male, SPF‐grade SD rats, aged 18 months, weighing 220–250 g, were purchased from the Experimental Animal Center of North Sichuan Medical College (animal use permit number: SYXK(Chuan)2018‐076). This experiment was reviewed and approved by the Animal Ethics Committee of North Sichuan Medical College (approval number: 2023097) and complied with national animal protection guidelines. All surgical procedures were performed under anesthesia, and every effort was made to minimize pain, suffering, and mortality. An animal model of age‐related tendinopathy was established by local injection of type I collagenase into the Achilles tendon of aged rats [40]. First, type I collagenase was prepared as a 50 mg/mL solution. Fifty microliters were injected locally into the body of the rat Achilles tendon. The senile tendinopathy model was established after 3 days. The rats were randomly divided into four groups. The PBS group received a local injection of only 50 µL of PBS into the Achilles tendon. The Ctrl group received 50 µL of type I collagenase locally injected into the Achilles tendon without any other intervention. The PVA/HPSe group received a local injection of 50 µL of type I collagenase and 50 µL of PVA/HPSe hydrogel. Finally, the PVA/HPSe@LiPo group received 50 µL of type I collagenase followed by 50 µL of the PVA/HPSe@LiPo hydrogel composite system. The groups were then assessed after 3, 4, and 5 weeks.

### 4.13 Histopathological Examination and Quantitative Tissue Analysis of Tendon Tissue

Macroscopic evaluations of the repaired tendon were performed at 3 weeks postoperatively, during which the Achilles tendon was fully exposed and photographed. Tissue staining and biosafety evaluation, immunohistochemistry, and immunofluorescence were performed. Briefly, the tendon tissues were collected and immediately fixed in 10% formalin for 24 h, dehydrated with graded alcohol, paraffin‐embedded, and then histologically sectioned. H&E and Masson's trichrome staining were used to examine major functional organs such as the heart, liver, spleen, lungs, and kidneys, evaluating the scaffold's biosafety. Masson's trichrome staining was used to analyze collagen properties and arrangement. The tendon tissue underwent Bonar scoring as previously published. Immunohistochemistry and immunofluorescence were used to analyze protein expression levels in the tendons. Briefly, immunohistochemical sections were incubated overnight at 4°C with primary antibodies such as anti‐Col 1 and anti‐TNC (Abcam, USA), anti‐OCN (Abcam, USA) followed by incubation with horseradish peroxidase‐conjugated secondary antibodies (Abcam, USA). Immunofluorescence sections were incubated with the primary antibodies, including anti‐CD68 and anti‐CD206 (Abcam, USA), followed by incubation with FITC‐ or rhodamine‐conjugated secondary antibodies at room temperature for 1 h. The cell nuclei were stained with DAPI. The samples were observed and imaged using confocal fluorescence microscopy.

### 4.14 Biomechanical Testing and Movement Function Analysis

The Achilles tendon was clamped in the fixture (China‐Sansi Zongheng‐UTM4103). With a preload of 50 N, it underwent 20 cyclic tensile cycles within a load range of 0–15 N at a loading rate of 2 mm/min. The specimen's mechanical properties were calculated based on loss stress (N), stiffness (N/mm), and tensile modulus (MPa).

Rat footprint analysis involves filling a defined corridor (approximately 90 cm long and 20 cm wide) with white paper. The rats’ hind paws were then evenly coated with black ink, allowing it to freely traverse the path and leave footprints on the paper. The footprint areas were then recorded and measured using ImageJ software.

### 4.15 Statistical Analysis

All data were repeated three times and expressed as mean ± standard deviation (SD). Data analysis was performed using GraphPad Prism 10.1.1 software. Comparisons between groups were conducted using *t*‐tests or one‐way analysis of variance (ANOVA). Data that were not normally distributed were expressed as median and compared using nonparametric tests. *P* < 0.05 was considered statistically significant, while *P* ≥ 0.05 indicated no significant difference.

## Ethics Approval Statement

The experimental protocol was approved by the Animal Ethics Committee of North Sichuan Medical College, with the approval number 2025008, in accordance with national animal protection guidelines. All necessary measures were taken to ensure the animals’ welfare, including anesthesia during procedures and efforts to minimize pain, suffering, and mortality.

## Patient Consent Statement

The experiment does not involve patients.

## Conflicts of Interest

The authors declare no conflicts of interest.

## Supporting information




**Supporting File**: advs74660‐sup‐0001‐SuppMat.docx

## Data Availability

The data that support the findings of this study are available from the corresponding author upon reasonable request.
