# Supplementary material for: Inhibiting cGAS‐STING to Preserve Mitochondrial–Nuclear Communication and Stemness in Young Tendon Stem Cells: A Hydrogel Strategy against Age‐Related Tendinopathy
Source: Adv Sci (Weinh). 2026 Mar 2;13(26):e20941. doi: 10.1002/advs.202520941 (PMC13159157; doi:10.1002/advs.202520941)
Supplement: Supplementary file 1 — Supporting File: advs74660‐sup‐0001‐SuppMat.docx [file ADVS-13-e20941-s001.docx]

**Supporting Information**

**Inhibiting cGAS-STING to Preserve Mitochondrial-Nuclear Communication and Stemness in Young Tendon Stem Cells: A Hydrogel Strategy against Age-Related Tendinopathyt**


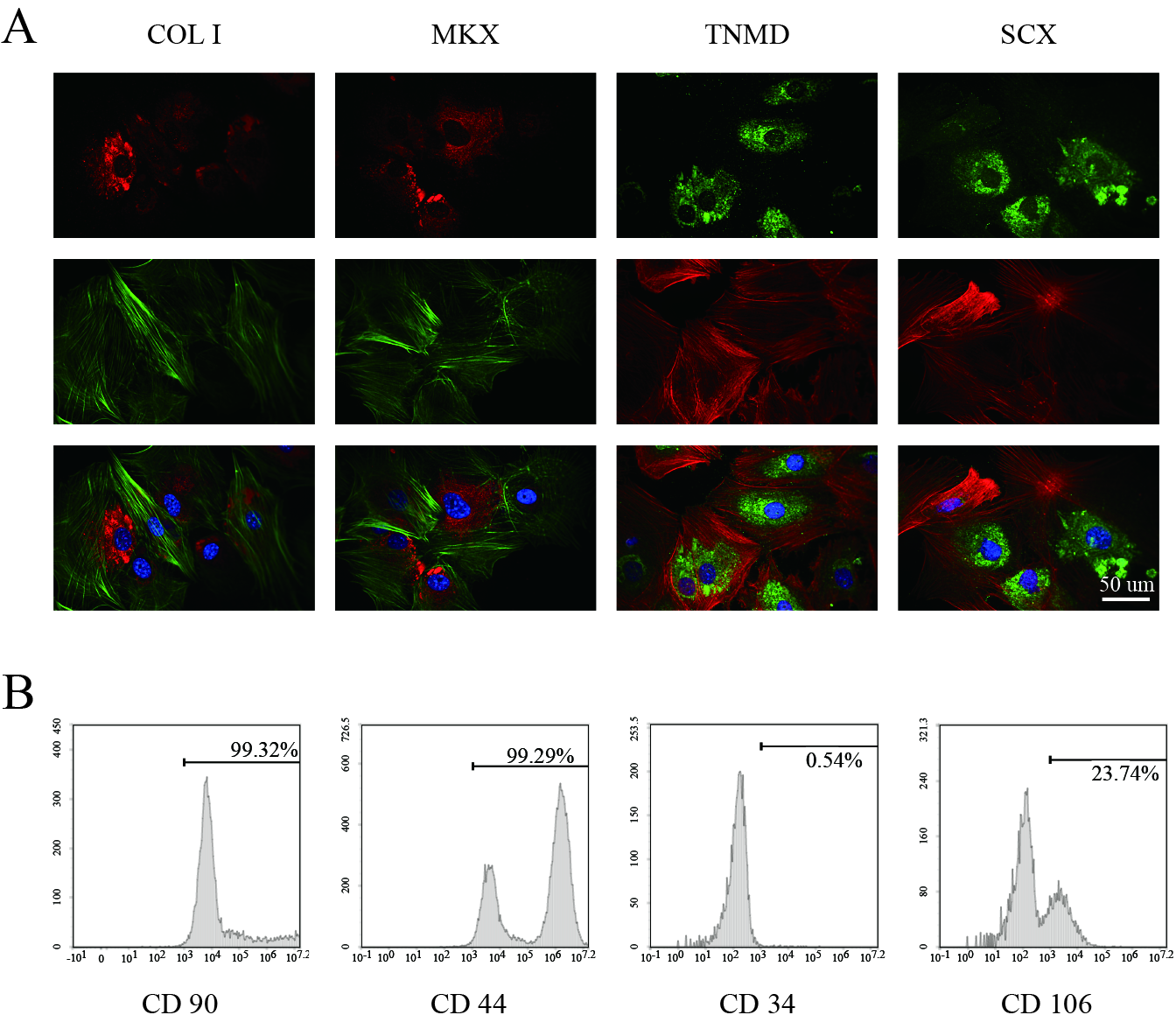


**Figure S1**. Characterization of TDSCs. A) Immunofluorescence staining images of tendon markers (COL I, TNMD, MKX, SCX) in isolated TDSCs. Cell nuclei stained with DAPI; cytoskeleton stained with Phalloidin. B) Flow cytometric characterization of TDSCs.


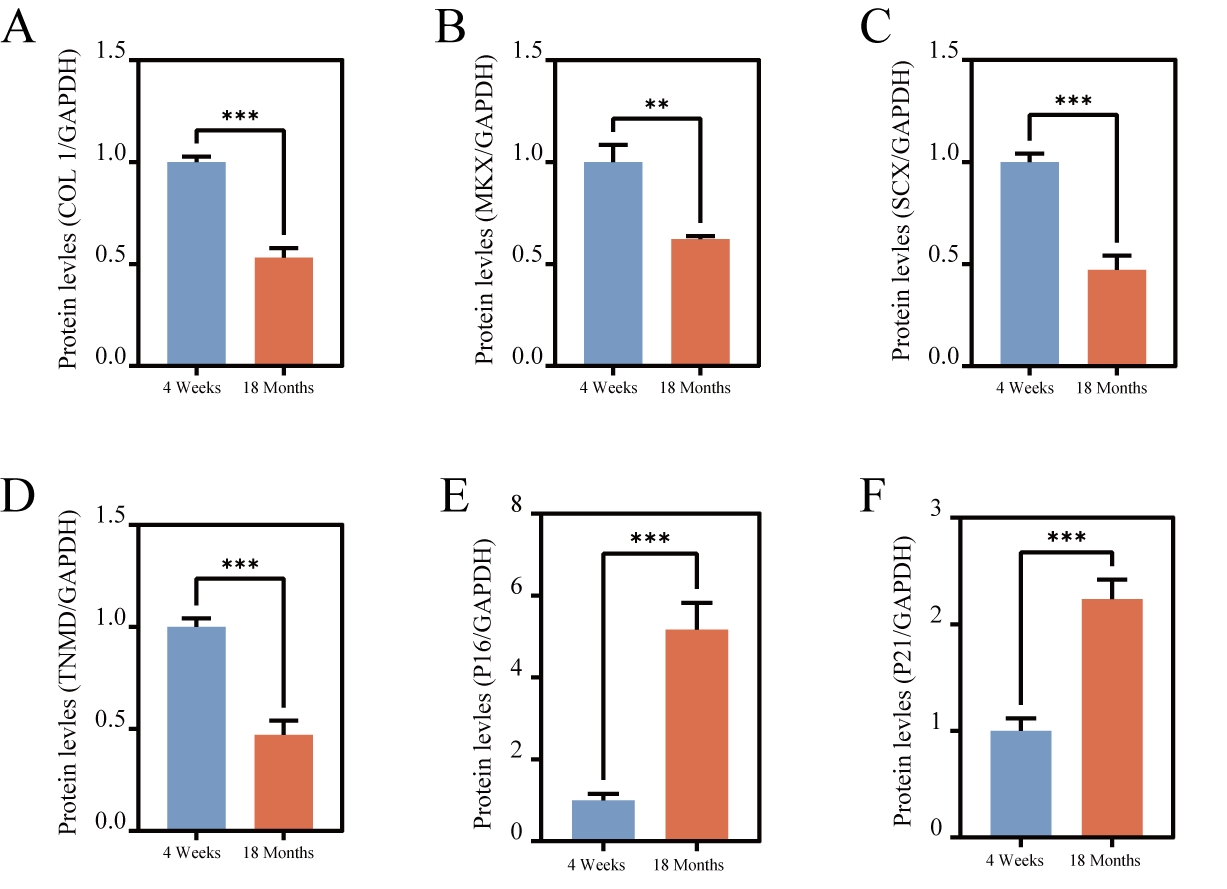


**Figure S2**. Gray values of COL 1, MKX, SCX, TNMD, P16, P21. (*n* = 3 per group) Data are expressed as mean ± SD. (* *p* < 0.05; ** *p* < 0.01; *** *p* < 0.001)

**Figure S3**. Schematic Diagram of the Synthesis of DSPE-PEG2K-Mal-CLHERHLNNN.


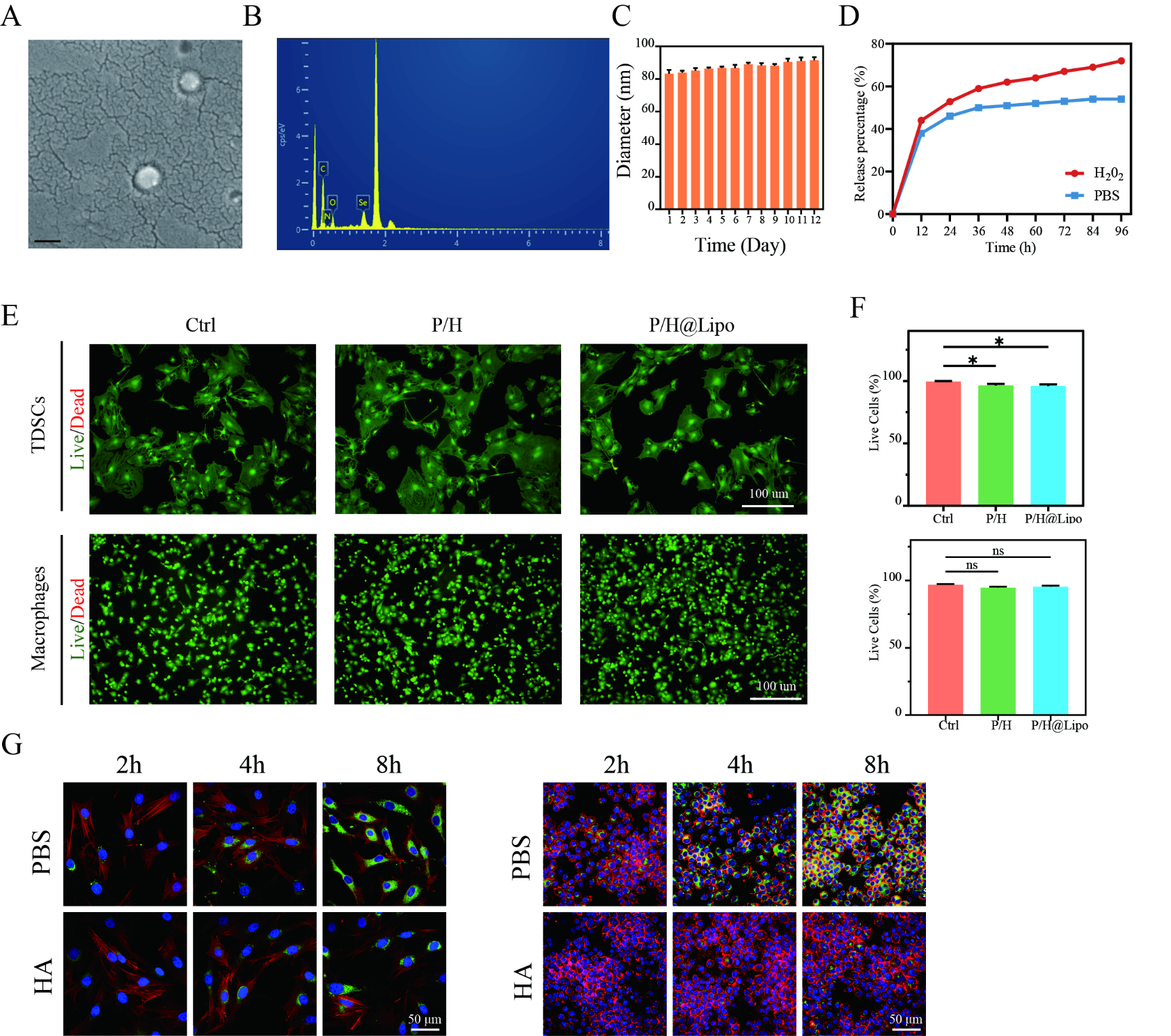


**Figure S4**. Scanning Electron Microscopy, Stability, Degradation, and Biocompatibility of HPSe in the P/H@Lipo hydrogel system. A, B) SEM images and electrostatic mapping of HPSe. C) Stability of HPSe. D) Degradation of P/H@Lipo in H₂O₂ and PBS solutions. E, F) Alcian blue staining and live/dead cell ratios after co-culture of P/H@Lipo hydrogel system with TDSCs and macrophages. G) Flow cytometry and immunofluorescence analysis of TDSCs and macrophages co-cultured with the hydrogel system after exposure to HA solution (with or without blocking), at different time points (green: coumarin 6-labeled HPSe; nuclei stained with DAPI; cytoskeleton stained with phalloidin). Data are expressed as mean ± SD. (*n* = 3 per group) Data are expressed as mean ± SD. (* *p* < 0.05; ** *p* < 0.01; *** *p* < 0.001)


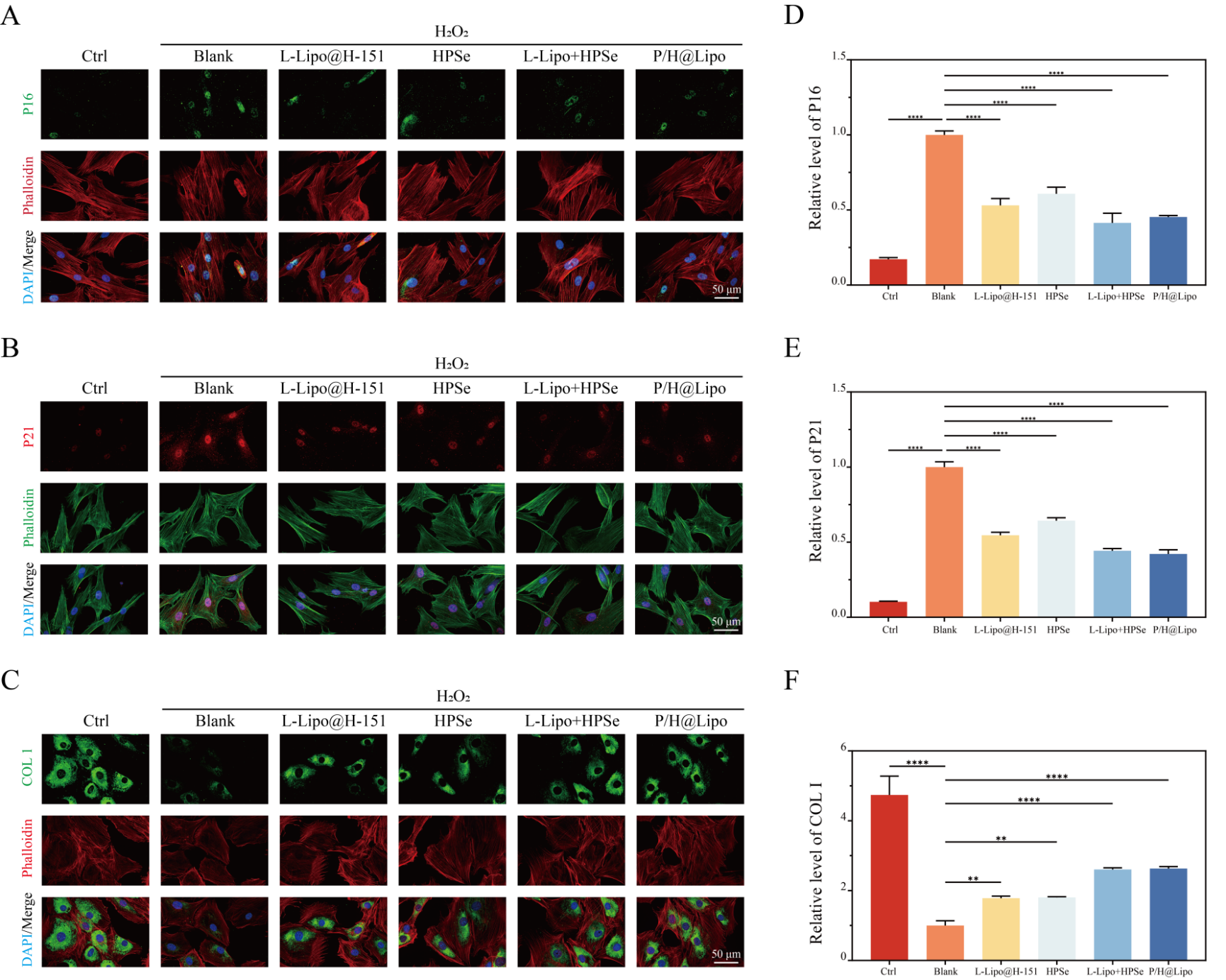


**Figure S5**. L-Lipo@H-151, HPSe, L-Lipo@H-151+HPSe, and P/H@Lipo Mitigated Senescence and Maintained the Function of Young TDSCs. (A, B) Immunofluorescence staining for p16, p21, and COL1 in TDSCs. Cell nuclei stained with DAPI; cytoskeleton stained with Phalloidin. D, E, F) Semi-quantitative analysis of immunofluorescence. Data are expressed as mean ± SD. (*n* = 3 per group) (* *p* < 0.05; ** *p* < 0.01; *** *p* < 0.001)


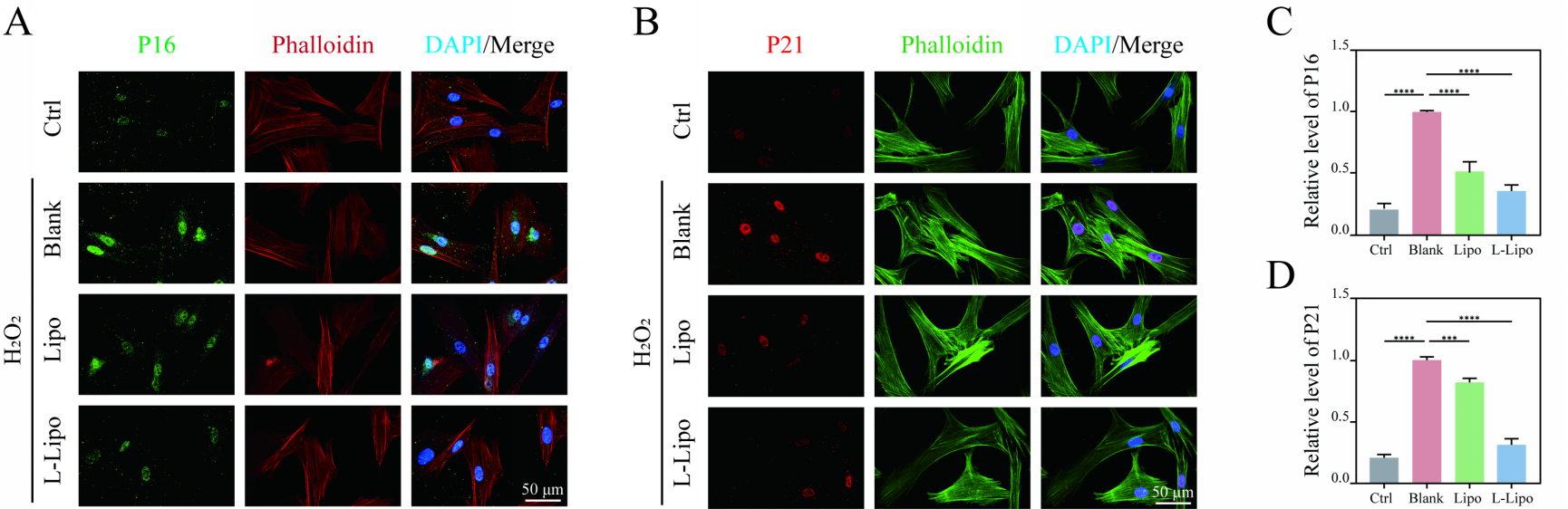


**Figure S6.** Lipo and L-Lipo Attenuate Aging in Young TDSCs in Vitro. A, B) Immunofluorescence staining of P16 and P21 in TDSCs. Cell nuclei stained with DAPI; cytoskeleton stained with Phalloidin. C, D) Corresponding semi-quantitative analysis. (*n* = 3 per group) Data are expressed as mean ± SD. (* *p* < 0.05; ** *p* < 0.01; *** *p* < 0.001)


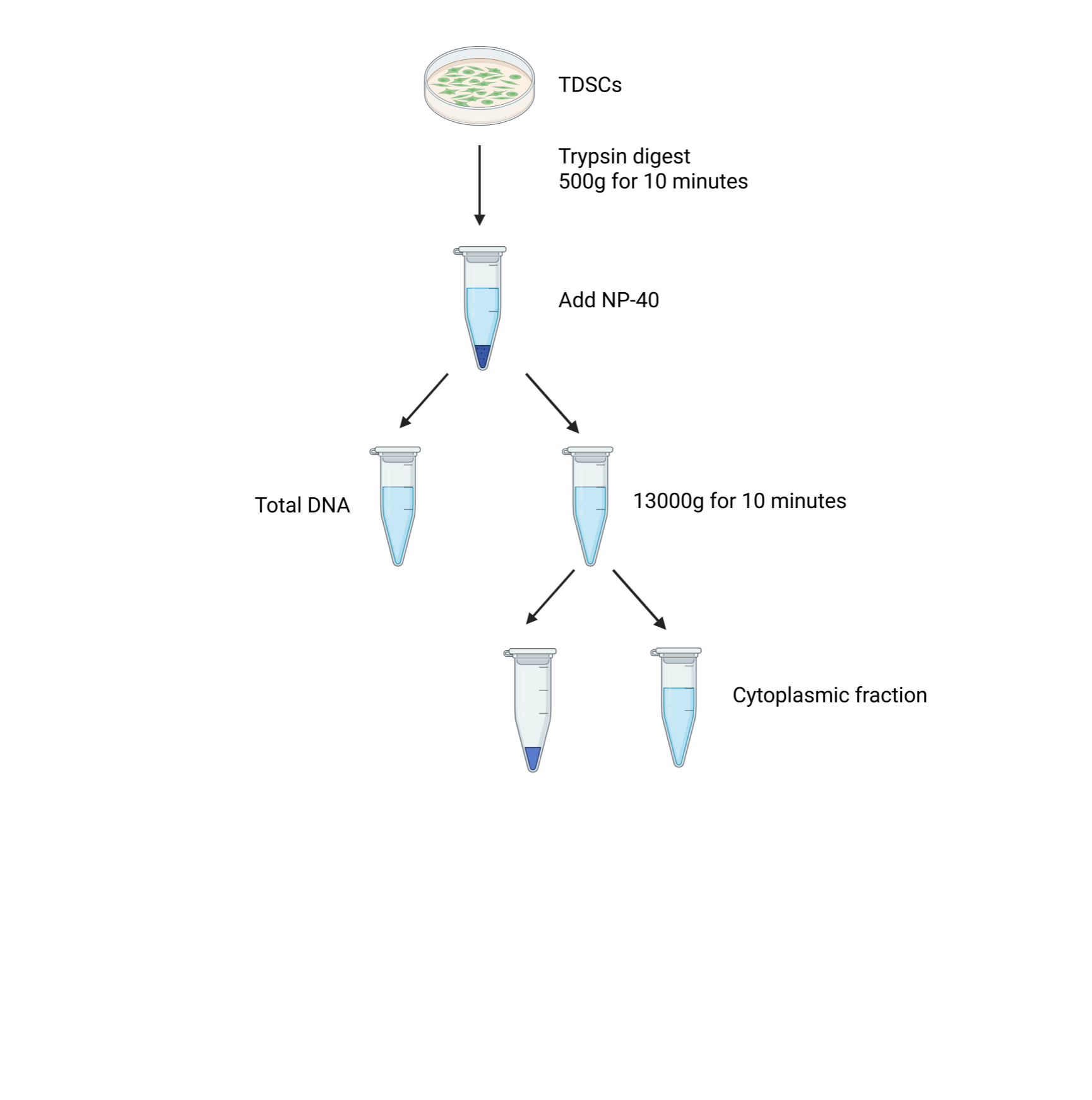
**Figure S7**. Schematic Diagram of the Steps for Extracting Total Cellular DNA and Cytoplasmic DNA.


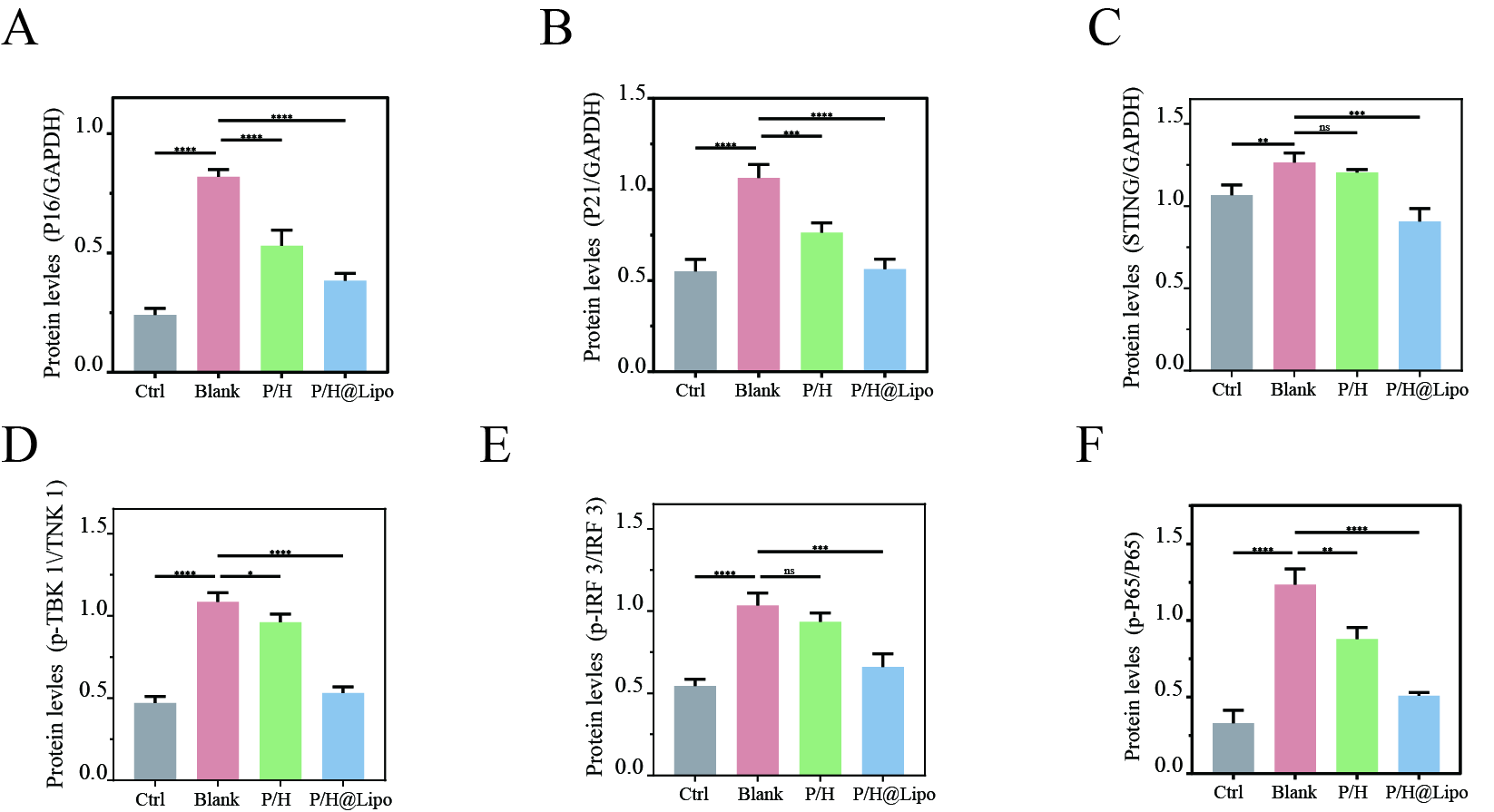


**Figure S8**. Gray Values of P16, P21, and STING Pathway-related Proteins. A–F) Semi-quantitative analysis of gray values from Western blots of P16, P21, and STING pathway-related proteins. G) Western blot images of Bax and Bcl-2 proteins. H, I) Quantitative analysis of gray values for Bax and Bcl-2 proteins. (*n* = 3 per group) Data are expressed as mean ± SD. (* *p* < 0.05; ** *p* < 0.01; *** *p* < 0.001)


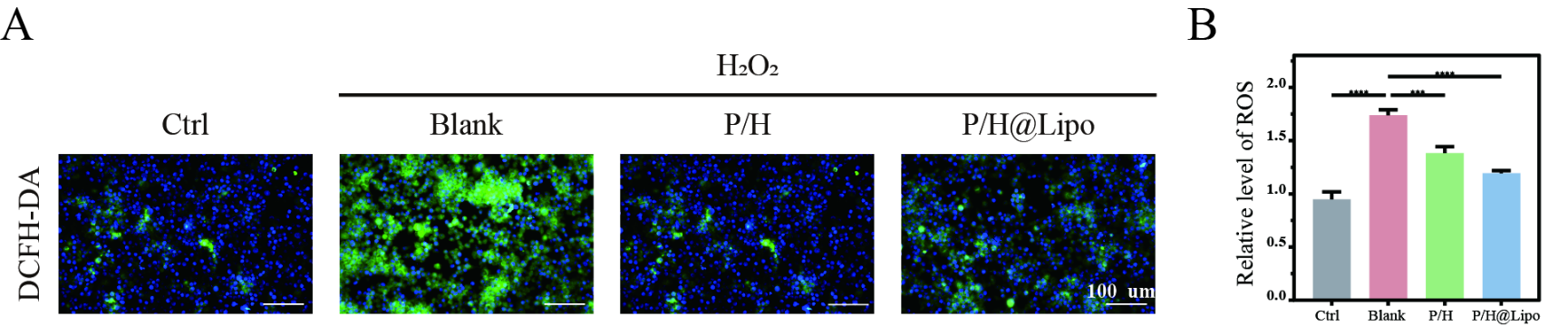


**Figure S9**. DCFH-DA Fluorescence Staining of Macrophages. A, B) DCFH-DA staining images and relative fluorescence intensity of macrophages. Data are expressed as mean ± SD. (* p < 0.05; ** p < 0.01; *** p < 0.001)


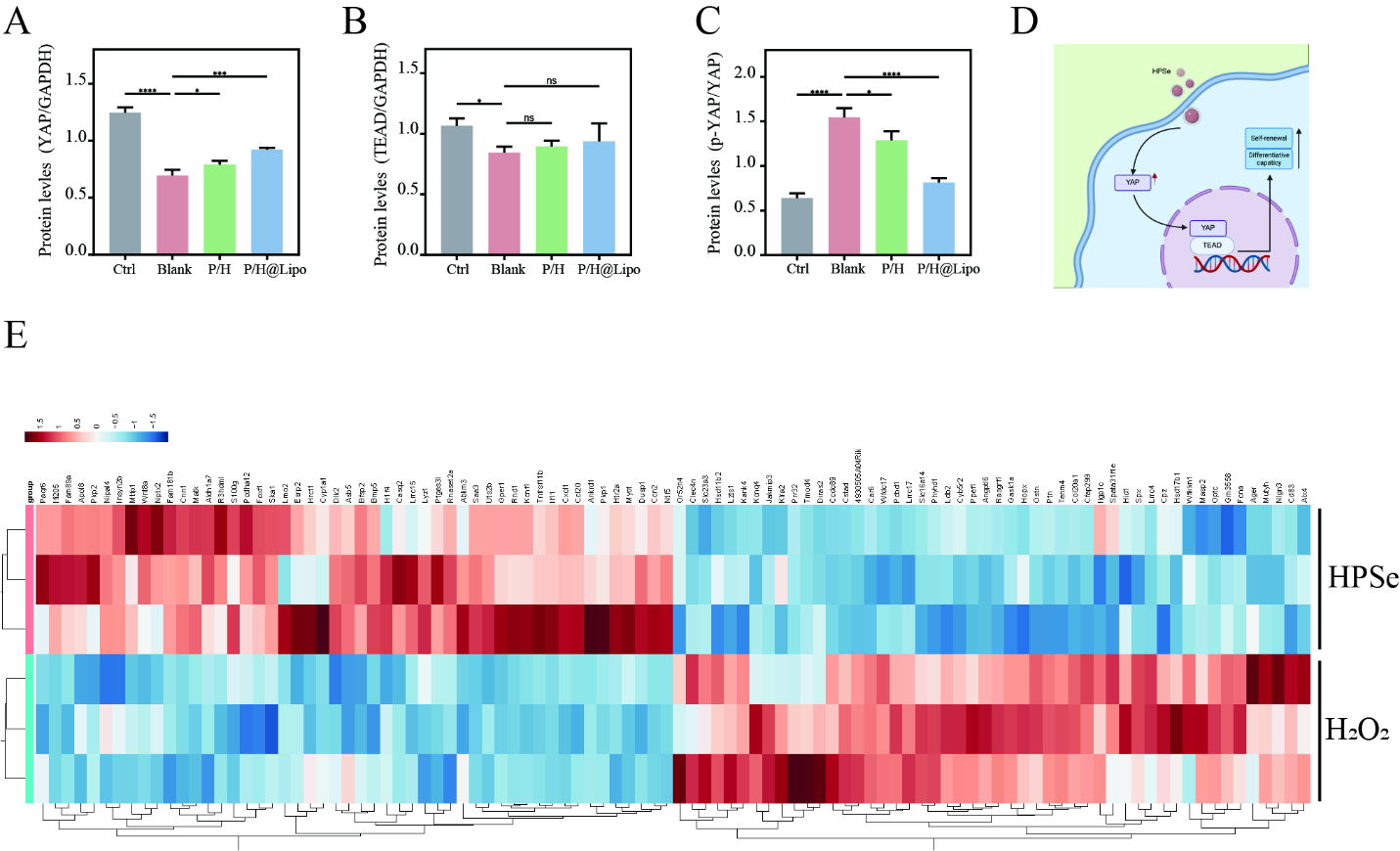


**Figure S10**. Analysis of Hippo Pathway Protein Immunoblot Grayscale Values, Schematic Diagram of HPSe Action, and Expression Heatmap of Differentially Regulated Genes. (*n* = 3 per group) Data are expressed as mean ± SD. (* *p* < 0.05; ** *p* < 0.01; *** *p* < 0.001)


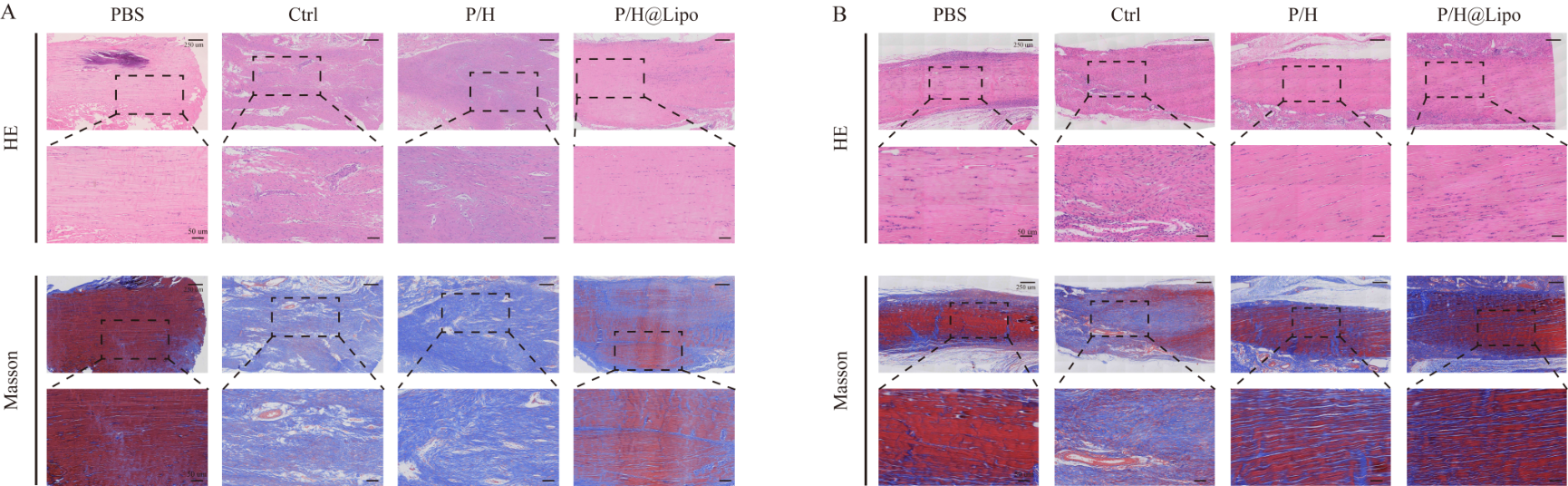


**Figure S11**. Histological Examination of Age-related Tendon Lesions at 4 and 5 weeks post-injury. A) H&E staining and Masson staining of tendons from different groups at 4 weeks post-injury. B) H&E staining and Masson staining of tendons from different groups at 5 weeks post-injury.


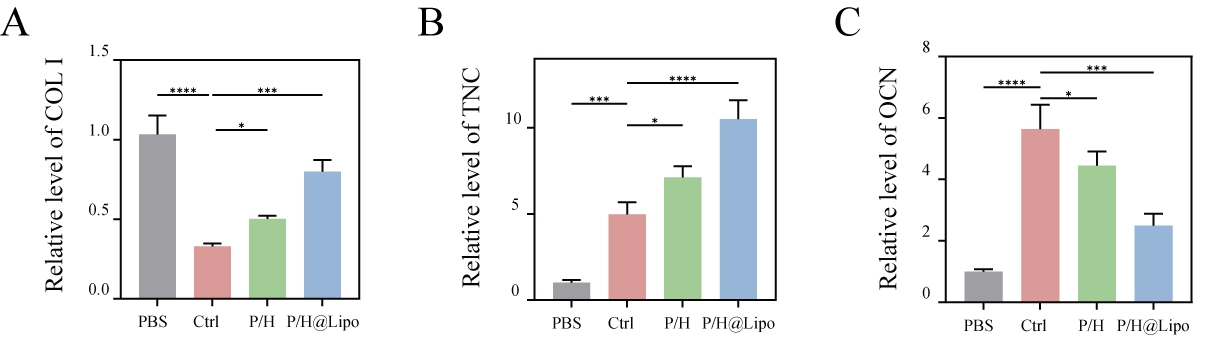


**Figure S12**. Histological Examination of Age-related Tendon Lesions at 4 and 5 weeks post-injury. A) H&E staining and Masson staining of tendons from different groups at 4 weeks post-injury. B) H&E staining and Masson staining of tendons from different groups at 5 weeks post-injury. (*n* = 3 per group) Data are expressed as mean ± SD. (* *p* < 0.05; ** *p* < 0.01; *** *p* < 0.001)


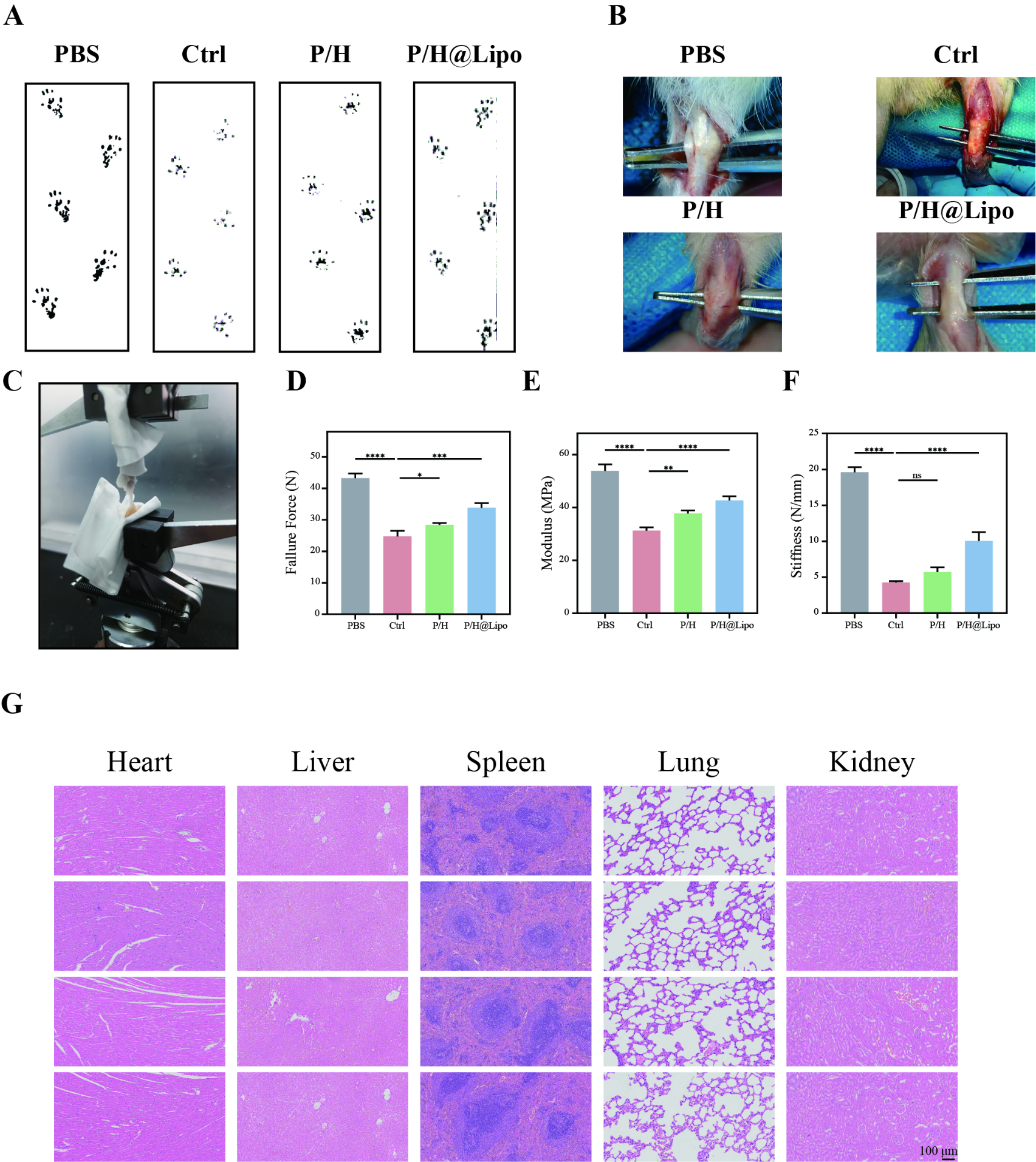


**Figure S13**. Gait analysis, Tendon Biomechanical Testing, and Biosafety Evaluation in Rats Across Different Treatment Groups. A) Gait patterns in rats from different groups. B) Tendon tissue images in rats from different groups. C) Schematic of tendon biomechanical testing. C–F) Testing of tendon yield stress, Young's modulus, and stiffness. G) H&E sections of heart, liver, spleen, lung, and kidney from different treatment groups. Data are expressed as mean ± SD. (* *p* < 0.05; ** *p* < 0.01; *** *p* < 0.001)

**Table S1**. Primer Sequences for for qRT-PCR.

| Gene | Sequences |
| --- | --- |
| *mtDNA -ND1* | 5′-TCCGAGCATCTTATCCACGC-3’ (Forward) |
| *mtDNA -ND1* | 5′-GTATGGTGGTACTCCCGCTG-3’ (Reverse) |
| *18S* | 5′-TAGAGGGACAAGTGGCGTTC-3’(Forward) |
| *18S* | 5′-CGCTGAGCCAGTCAGTGT-3’ (Reverse) |
